# A synbiotic intervention modulates meta-omics signatures of gut redox potential and acidity in elective caesarean born infants

**DOI:** 10.1186/s12866-021-02230-1

**Published:** 2021-06-25

**Authors:** Christophe Lay, Collins Wenhan Chu, Rikky Wenang Purbojati, Enzo Acerbi, Daniela I. Drautz-Moses, Paola Florez de Sessions, Song Jie, Eliza Ho, Yee Jiun Kok, Xuezhi Bi, Shuwen Chen, Shi Ya Mak, Mei Chien Chua, Anne E. N. Goh, Wen Chin Chiang, Rajeshwar Rao, Surasith Chaithongwongwatthana, Nipon Khemapech, Voranush Chongsrisawat, Rocio Martin, the JULIUS Study Group, Yanqing Koh, Sachin R. Lohar, Ivan Chin Hin Tan, Wong Anng Anng, Chen Jie, Nana Bartke, Kaouther Ben-Amor, Ingrid B. Renes, Fiona Wong, Guus Roeselers, Ying Swan Ho, Martin L. Hibberd, Stephan C. Schuster, Jan Knol

**Affiliations:** 1 Danone Nutricia Research, Singapore, Singapore; 2 Genome Institute of Singapore, Singapore, Singapore; 3 Singapore Centre For Environmental Life Sciences Engineering (SCELSE), Nanyang Technological University, Singapore, Singapore; 4 Bioprocessing Technology Institute, Singapore, Singapore; 5 KK Women’s and Children’s Hospital, Singapore, Singapore; 6 King Chulalongkorn Memorial Hospital, Faculty of Medicine, Chulalongkorn University, Bangkok, Thailand; 7 Danone Nutricia Research, Utrecht, The Netherlands; 8 London School of Hygiene and Tropical Medicine, London, UK; 9 Wageningen University, Wageningen, The Netherlands

**Keywords:** Microbiome, Infant, C-section, Synbiotics

## Abstract

**Background:**

The compromised gut microbiome that results from C-section birth has been hypothesized as a risk factor for the development of non-communicable diseases (NCD). In a double-blind randomized controlled study, 153 infants born by elective C-section received an infant formula supplemented with either synbiotic, prebiotics, or unsupplemented from birth until 4 months old. Vaginally born infants were included as a reference group. Stool samples were collected from day 3 till week 22. Multi-omics were deployed to investigate the impact of mode of delivery and nutrition on the development of the infant gut microbiome, and uncover putative biological mechanisms underlying the role of a compromised microbiome as a risk factor for NCD.

**Results:**

As early as day 3, infants born vaginally presented a hypoxic and acidic gut environment characterized by an enrichment of strict anaerobes (Bifidobacteriaceae). Infants born by C-section presented the hallmark of a compromised microbiome driven by an enrichment of Enterobacteriaceae. This was associated with meta-omics signatures characteristic of a microbiome adapted to a more oxygen-rich gut environment, enriched with genes associated with reactive oxygen species metabolism and lipopolysaccharide biosynthesis, and depleted in genes involved in the metabolism of milk carbohydrates. The synbiotic formula modulated expression of microbial genes involved in (oligo)saccharide metabolism, which emulates the eco-physiological gut environment observed in vaginally born infants. The resulting hypoxic and acidic milieu prevented the establishment of a compromised microbiome.

**Conclusions:**

This study deciphers the putative functional hallmarks of a compromised microbiome acquired during C-section birth, and the impact of nutrition that may counteract disturbed microbiome development.

**Trial registration:**

The study was registered in the Dutch Trial Register (Number: 2838) on 4th April 2011.

**Supplementary Information:**

The online version contains supplementary material available at 10.1186/s12866-021-02230-1.

## Background

The first 1000 days of life is recognized as an important window to nurture child health and development [1]. An increasing body of evidence indicates that a compromised microbiome in early life is a risk factor for the development of non-communicable diseases [2–10]. A wealth of epidemiological data has described associations between prenatal or postnatal exposure to antibiotics, C-section birth and immune and metabolic health [11–14]. As a result, early life antibiotics exposure or C-section birth have been implicated as risk factors for asthma, eczema, obesity and type 2 diabetes [12, 15–18]. A recent population cohort study of 7.17 million births described an association between birth by C-section and infection related hospitalisation in early childhood [19]. It appears that a perturbation of the transmission of the maternal microbiome, as well as a compromised development of the infant microbiome because of antibiotic exposure or C-section birth have long-term health consequences. Several studies depicted a delayed colonization by “keystone taxa”, *Bifidobacterium* and *Bacteroides* in Caesarean born infants [20–27].

These keystone colonizers are acquired vertically from the maternal microbiome [28] and have evolved the genomic capability to metabolize human milk oligosaccharides (HMOs) and play a pivotal role in immune programming [29, 30]. These biological traits equip them to maintain a robust symbiosis with their human host. Increased abundance of members of the Enterobacteriaceae, such as *Klebsiella*, has been described in the gut of infants born by C-section, however little is known about their functional role in the infant gut and impact on host health [21, 22, 27, 31].

**Fig. 1:**
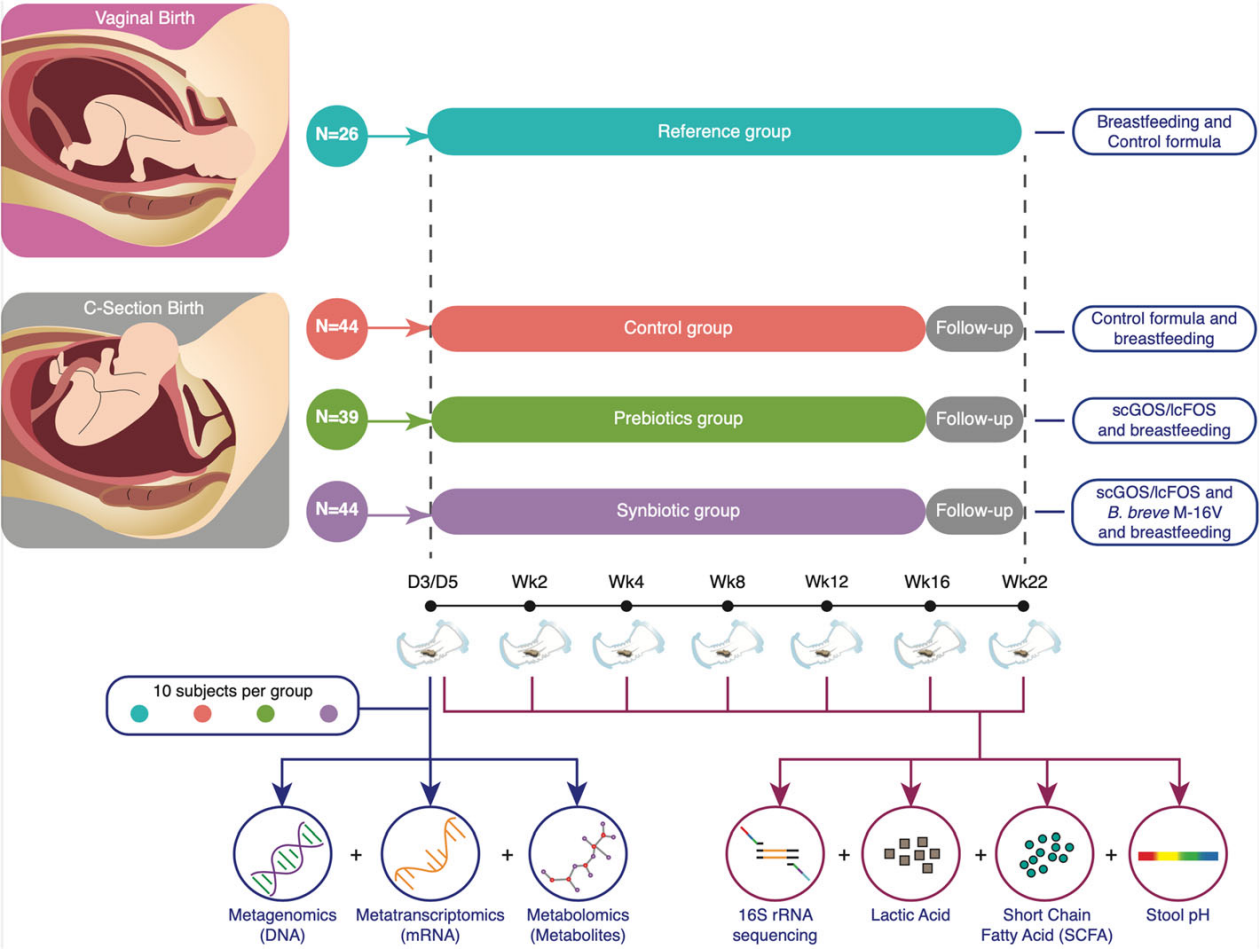
Infographic depicting the study design. Depiction of the multi-omics approach used to investigate the functional impact of mode of delivery and nutritional interventions on the infant gut microbiome. In this study, the infants were mixed fed. Subjects from each group of infants born by elective C-section (Synbiotic, Prebiotics and Control) received the study product corresponding to their allocated group in addition to breastfeeding. The group of vaginally born infants or Reference group was also mixed fed and a control formula was provided. This allowed us to ascertain that observed differences between study arms and the reference group resulted from mode of delivery and nutritional interventions. In this study, the number of subjects per group whose faecal genomic DNA was available for 16S rRNA sequencing, was as follows: Synbiotic group (*n* = 44), Prebiotic group (*n* = 39), Control group (*n* = 44) and Reference group (*n* = 26) and those numbers are reflected in Figure 1

A study suggested that swabbing infants born by C-section immediately after birth with vaginal secretions could partially restore the lack of maternal microbiota transmission [32]. However, concerns about the risk of infection associated with this practice have been raised [33]. Chua and colleagues demonstrated that a specific synbiotic, a combination of short-chain galacto-oligosaccharides (scGOS) and long-chain fructo-oligosaccharides (lcFOS), and *Bifidobacterium breve* M-16 V, restored the delayed colonization by *Bifidobacterium* in elective C-section born infants [18]. This nutritional intervention was likely associated with a lower incidence of eczema/atopic dermatitis observed among infants receiving the synbiotic formula [18]. Here, the fecal samples collected within the aforementioned clinical trial were analysed through a multi-omics approach (16S rRNA gene amplicon sequencing, shotgun metagenomics, metatranscriptomics and metabolomics) (Fig. 1). These analyses revealed that the compromised microbiome acquired during elective C-section birth reflects a microbiome adapted to a more oxidative environment characterised by functional signatures of reactive oxygen species metabolism, biosynthesis of lipopolysaccharides and the absence of detection of genes, transcripts involved in the metabolism of milk carbohydrates. In this paper, we provide molecular signatures that indicate a significant difference in the developing microbial ecosystem of C-section versus vaginally born infants. We demonstrate that a specific synbiotic (a combination of scGOS/lcFOS and *Bifidobacterium breve* M-16 V) intervention prevents in elective C-section born infants the establishment of a compromised microbiome in the first days of life.

## Results

A previous study by Chua and colleagues demonstrated that a synbiotic intervention could prevent the delayed intestinal colonization by *Bifidobacterium* sp. in elective C-section born infants [18]. In the current study, faecal samples from these infants were subjected to multi-omics approach uncovering functional features of a compromised microbiome acquired during elective C-section birth. The aim was to (1) elucidate functional characteristics of a compromised microbiome, (2) form hypotheses on the mechanisms by which a compromised microbiome can influence disease risk and (3) investigate the functional role of a specific synbiotic (scGOS/lcFOS and *Bifidobacterium breve* M-16 V) in restoring a compromised microbiome.

### Description of the study population for feeding pattern and intrapartum antibiotic prophylaxis

In Singapore and Thailand, exclusive breastfeeding is rare and therefore the recruitment of elective C-section and vaginally born infants that are exclusively breastfed is challenging. In this study, most infants were mixed fed. Subjects from each group of infants born by elective C-section (Synbiotic, Prebiotics and Control) received the study product corresponding to their allocated group in addition to breastfeeding. The group of vaginally born infants or Reference group was also mixed fed and a control formula was provided [18]. This allowed us to ascertain that observed differences between study arms and the reference group resulted from mode of delivery and nutritional interventions. In our study, all the infants born by elective C-section were exposed to intrapartum antibiotic prophylaxis (IAP), which is the common clinical practice in Singapore and Thailand. Among the infants from the vaginally born reference group (*n* = 30), two infants were exposed to IAP. They were excluded from this multi-omics study since IAP was considered as a potential confounder. Although comparison with additional study groups such as infants born by elective C-section without IAP exposure and infants born vaginally with IAP exposure could have provided additional insight, this was not investigated in this study.

### Sequencing and metabolomic output

In this study, the number of subjects per group whose faecal genomic DNA was available for 16S rRNA sequencing, was as follows: Synbiotic group (*n* = 44), Prebiotics group (*n* = 39), Control group (*n* = 44) and Reference group (*n* = 26) (Supplementary Table S1). For data analysis and interpretation, these intervention groups were labelled as Synbiotic group, Prebiotics group, Control group and Reference group. Faecal genomic DNA (943 samples) were available for 16S rRNA sequencing from 153 subjects (Supplementary Table S1). The average number of sequencing quality-passed reads mapping to the 16S rRNA Greengenes global rRNA database were 1,267,887 ± 667,938 for the Synbiotic group; 1,233,575 ± 651,949 for the Prebiotics group; 1,098,486 ± 556,112 for the Control group and 1,197,828 ± 637,407 for the Reference group (Supplementary Table S2).

The metagenomics and metatranscriptomics data were generated from a subset of the stool samples collected at day 3 and/or day 5 (Fig. 1). The samples were selected randomly from 10 subjects per group from the clinical study’s biobank. The metagenomics sequencing run produced, on average, 8.6 million paired-end reads per sample, or 4.3Gb data per-sample. After quality control and removal of host sequences, on average, 6,864,061 (79.7%) paired-end reads were used for downstream metagenomics analysis. At the end of the classification analysis, on average, 88.93% (66.78–94.97%) of the paired-end reads could be classified to the kingdom Bacteria. The metatranscriptomics sequencing run produced, on average, 11.9 million paired-end reads per sample, or 2.4Gb data per-sample. After quality control and removal of ribosomal RNA sequences, on average, 1,212,609 (10.12%) paired-end reads were categorized as non-ribosomal RNA reads and used for downstream metatranscriptomics analysis. The resulting analysis showed that, on average, 60.31% (40–75.28%) of the non-ribosomal RNA reads could be classified to the kingdom Bacteria.

The metabolomics data were generated from a subset of the stool samples collected at day 3 and/or day 5 (Fig. 1). The samples were selected randomly from 10 subjects per group from the clinical study’s biobank. The 10 samples per group were then pooled before metabolomics profiling. The metabolomics dataset represented four pools reflecting the intervention and reference groups.

### Delayed colonization by keystone colonizers in C-section born infants

**Fig. 2:**
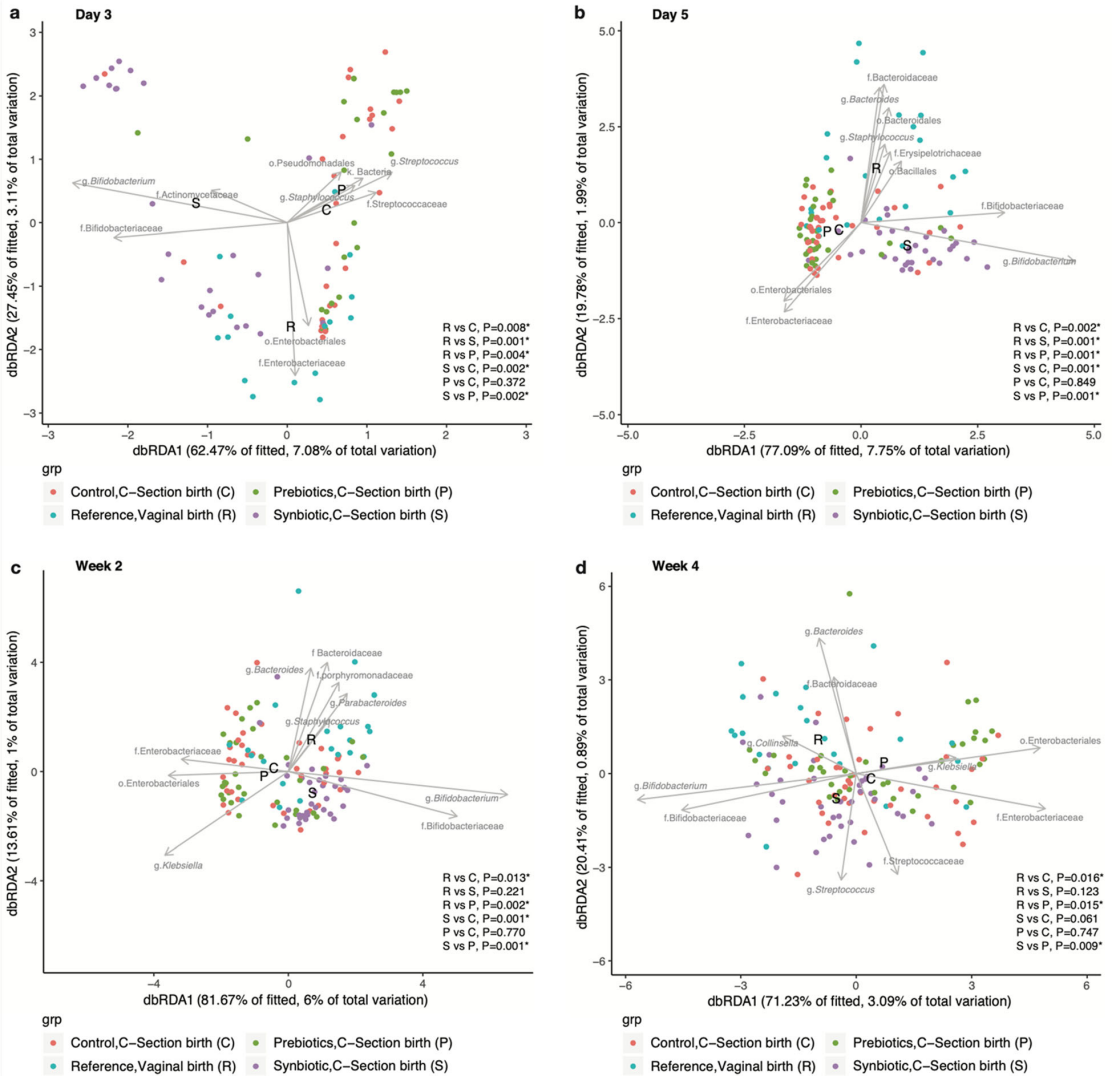
Distance based redundancy analysis (db-RDA) of infant gut microbiome. Subjects belonging to the intervention groups, Control C-section (C), Prebiotics C-section (P), Synbiotic C-section (S) and the Reference group (R) are indicated by the red, green, purple and blue circles respectively. The db-RDA axes describe the percentage of the fitted and total variation explained by each axis while being constrained on groups. Black arrow vectors indicate the weight and direction of 10 most important bacterial genera. The labels for the groups, Control C-section (C), Prebiotics C-section (P), Synbiotic C-section (S) and the Reference group (R) are located at the geometrical center of each intervention group. * represents *P* < 0.05 (PERMANOVA test)

**Fig. 3:**
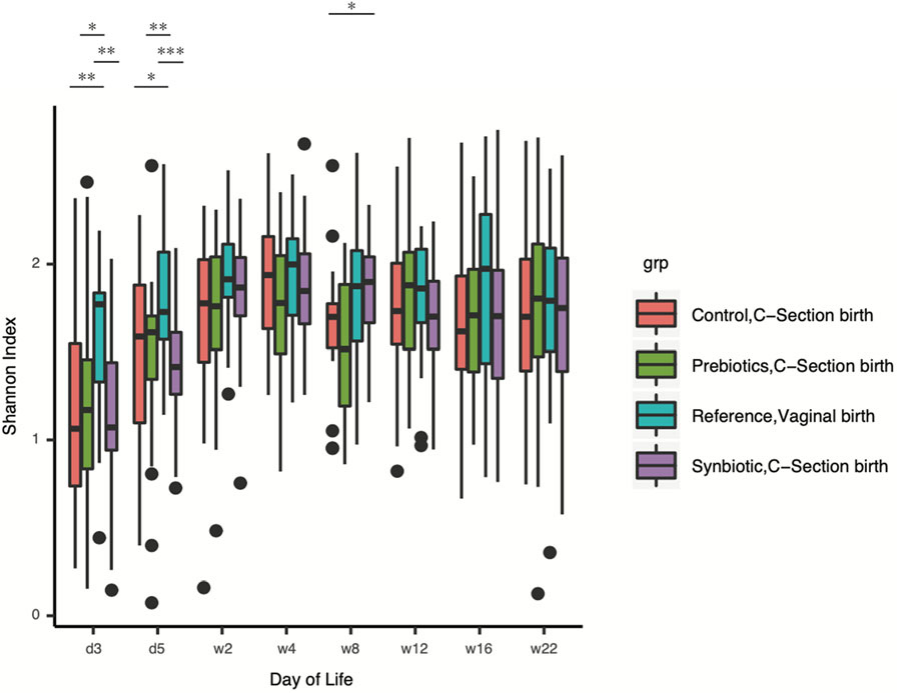
Species diversity per intervention group. Shannon index plot depicting the species diversity (Genus dataset) per intervention group from day 3 till week 22. Non-parametric Mann-Whitney U tests, *P* < 0.05, *P* < 0.01 and *P* < 0.001 are indicated by *, ** and *** respectively

16S rRNA data were used to determine the effect of mode of delivery on the development of the infant gut microbiota. We applied a constrained supervised ordination method, db-RDA and PERMANOVA to test the hypothesis that mode of delivery influences the pioneer bacterial colonization of the infant gut. The ordination plots (Fig. 2a and b) show that the first two components separate samples from the Control (C-section birth) and Reference (vaginal birth) groups collected at day 3 and 5. The bacterial communities of both groups of infants were compared across time points, a statistically significant difference was observed between day 3 and week 16 (Supplementary Table S3). Pairwise group comparisons between the Control and Reference groups showed that *Bifidobacterium* and *Bacteroides* were the predominant genera that separate both groups (Supplementary Fig. 1 and Tables S5 and S8). The abundance of members of Enterobacteriaceae was statistically significantly higher in the Control group compared to the Reference group at day 5 (*P* = 0.020) (Supplementary Tables S4 and S7). A lower species diversity was observed in the Control group compared to the Reference group at day 3 and day 5 (*P* = 0.008 and *P* = 0.037, respectively) (Fig. 3).

### Elective C-section birth is associated with a delayed establishment of strict anaerobes

**Fig. 4:**
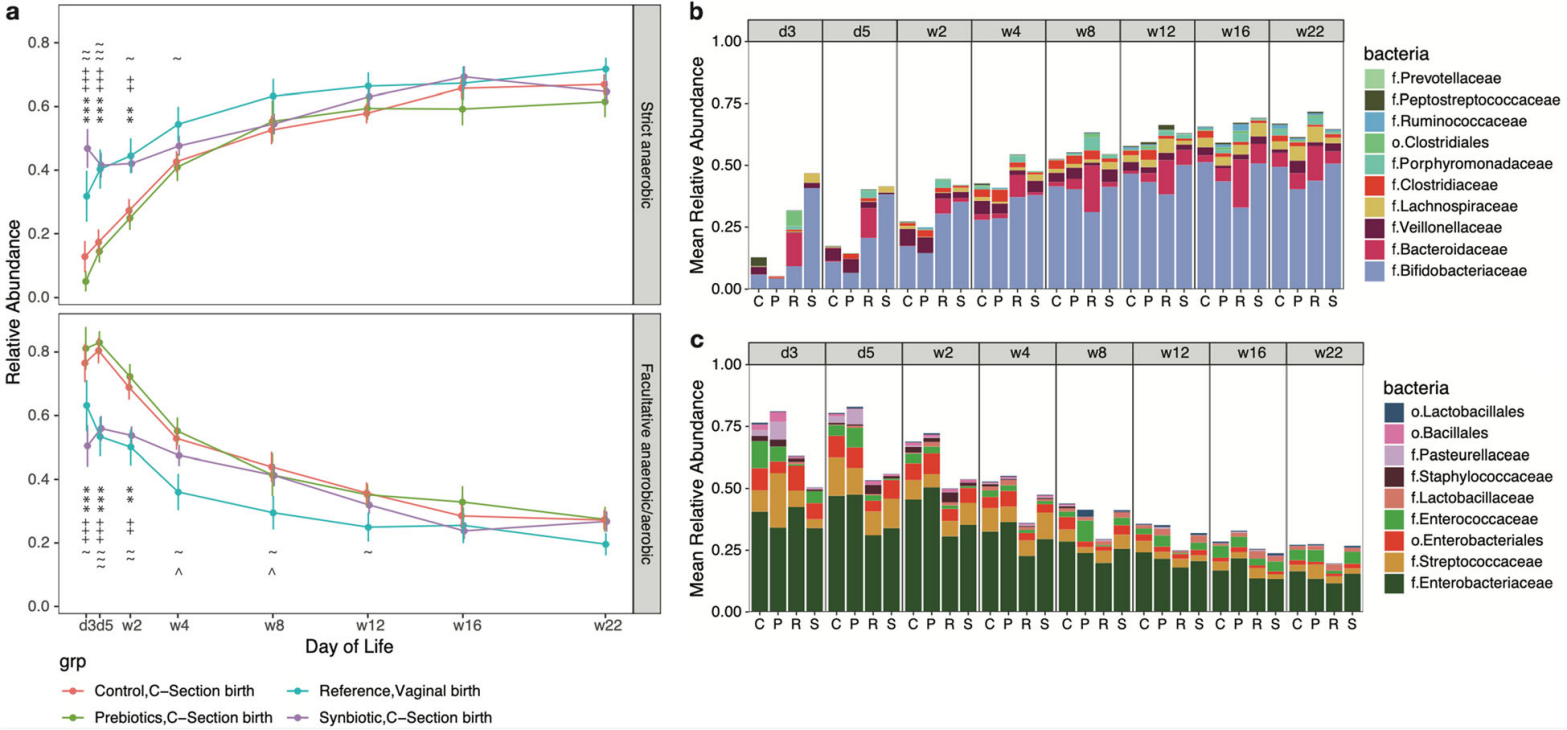
**a** Relative abundance of strict anaerobes and facultative anaerobes/aerobes. Line plots of the relative abundance of strict anaerobes and facultative anaerobes/aerobes from day 3 till week 22. Non-parametric Mann-Whitney U tests, *P* < 0.05, *P* < 0.01 and *P* < 0.001 are indicated by (Synbiotic vs Control: *, **, ***), (Synbiotic vs Prebiotic: +, ++, +++), (Synbiotic vs Reference, Vaginal Birth: ^, ^^, ^^^), (Reference, Vaginal Birth vs Control: ~, ~~, ~ ~ ~) respectively. **b** Relative abundance of strict anaerobes. Stacked bar plots of the relative abundance of the key bacterial groups at the Family level that contribute to the strict anaerobes from day 3 till week 22. **c** Relative abundance of the facultative anaerobes/aerobes. Stacked bar plots of the relative abundance of the key bacterial groups at the Family level that contribute to the facultative anaerobes/aerobes from day 3 till week 22

Differential abundances of pioneer colonizers between vaginally and C-section born infants could be translated to functional differences in metabolism. We hypothesized that the physiological environment of the gut differs between vaginally and C-section born infants. To test this hypothesis, we categorized the bacterial families into functional groups by determining their anaerobic/aerobic metabolism. The Bergey’s Manual of Systematics of Archaea and Bacteria was used as reference to predict the metabolic repertoire of those bacterial families listed in Supplementary Table S10 [34]. The relative abundance of those bacterial families was summed according to their functional groups (strict anaerobic, facultative anaerobic/ aerobic). Infants born vaginally presented an enrichment of members of Bifidobacteriaceae and Bacteroidaceae, characteristic of an anaerobic gut environment (Fig. 4a and b). In contrast, infants born by C-section (Control group) harbored more facultative anaerobes and aerobes, mainly represented by members of the Enterobacteriaceae (Fig. 4a and c) [34]. This may be characteristic of a more oxygen rich gut environment. Infants born by C-section featured a delayed colonization by strict anaerobes and this was statistically significantly different from the vaginally born infants between day 3 and week 4 (*P* = 0.002 and *P* = 0.041, respectively). We hypothesized that anaerobiosis was a key microbial metabolic trait among infants born vaginally, whereas aerobiosis was a key microbial metabolic trait among C-section born infants.

### A synbiotic intervention supports species diversity and *Bifidobacterium* abundance in elective C-section born infants

Multivariate analysis was used on 16S rRNA data to determine the relationship between the nutritional intervention and the bacterial colonization of the infant gut. The ordination plots (Fig. 2a, b and c) show that the taxonomic composition of the Synbiotic group is distinct from the Control group at day 3, day 5 and week 2 (Supplementary Table S3). Similar results were observed when we compared the Synbiotic and the Prebiotic groups between day 3 and week 4 (Supplementary Table S3). No statistically significant differences were observed between Prebiotic and Control groups. Pairwise group comparisons showed that *Bifidobacterium* was the main genus that separated the Synbiotic from the Control and Prebiotic groups (Supplementary Tables S5 and S8). Shannon Diversity analysis revealed no statistically significant difference between the Synbiotic and Control groups except at week 8 (*P* = 0.018) (Fig. 3). Across the whole intervention period, the bacterial community composition of the Control group was dissimilar to the Reference group. The dissimilarity between the Prebiotic and Reference groups was statistically significant between day 3 and week 4 (Supplementary Table S3). Using Shannon Diversity, a higher species diversity was observed in the Reference group compared to the Prebiotic and Synbiotic groups, and this was statistically significant at day 3 and day 5 (Reference versus Prebiotic, *P* = 0.018 and *P* = 0.009, respectively; Reference versus Synbiotic, *P* = 0.004 and *P* < 0.001, respectively) (Fig. 3). In contrast, no statistically significant differences in microbiota composition were detected between the Synbiotic and the Reference groups at week 2 and week 4 (Supplementary Table S3, Fig. 2c and d and Supplementary Fig. 2a and b).

### A synbiotic intervention restores molecular signatures that are indicative of low oxygen and low pH in elective C-section born infants

**Fig. 5:**
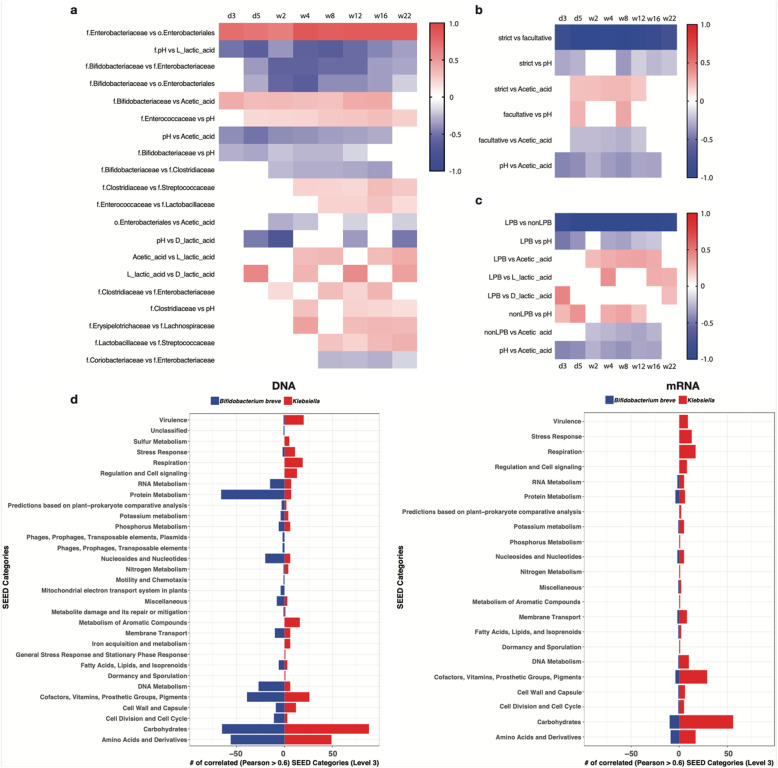
**a** Correlation between stool physiological parameters and the bacterial group abundances. Heat map showing the Spearman’s correlation coefficients between stool physiological parameters and the bacterial group abundances (Family level, 16S rRNA data) from day 3 till week 22. The Spearman’s rank correlation coefficients were determined by combining the biological datasets from all 4 groups. **b** Correlation between stool physiological parameters and the functional bacterial group abundances (strict and facultative anaerobes). Heat map showing the Spearman’s correlation coefficients between stool physiological parameters and the functional group abundances (strict and facultative anaerobes, 16S rRNA data) from day 3 till week 22. The Spearman’s rank correlation coefficients were determined by combining the biological datasets from all 4 groups. **c** Correlation between stool physiological parameters and the functional bacterial group abundances (Lactic acid bacteria determined with The Bergey’s Manual of Systematics of Archaea and Bacteria, supplementary Table S10). Heat map showing the Spearman’s correlation coefficients between physiological parameters and the functional group abundances (Lactic Acid Bacteria(LAB), 16S rRNA data) from day 3 till week 22. The Spearman’s rank correlation coefficients were determined by combining the biological datasets from all 4 groups. **d** Correlations between *Bifidobacterium breve*/*Klebsiella* at metagenomics and metatranscriptomics levels. Bar plots depicting correlations between *Bifidobacterium breve*/*Klebsiella* and functional genes (DNA and mRNA, SEED data)

We observed that initially the composition of the gut microbiota in the Synbiotic group differed from the Reference group at day 3 and day 5 (Synbiotic versus Reference, *P* = 0.001 and P = 0.001, respectively) (Supplementary Table S3, Fig. 2a and b). We demonstrated that after a certain number of days of supplementation, the Synbiotic and Reference groups did not differ in microbiota composition at week 2 and week 4 (Supplementary Table S3, Fig. 2c and d). We then tested the hypothesis that both groups shared a similar microbial metabolism. The Synbiotic supplementation resulted in an anaerobic gut microbial environment characterized by an enrichment of Bifidobacteriaceae (Fig. 4a and b) as early as day 3. The formation of the strict anaerobic environment was similar between the Synbiotic and Reference groups. In contrast, infants from the Control and Prebiotic groups presented a delayed colonization by strict anaerobes when compared to the Synbiotic group, and this was statistically significantly different between day 3 and week 2 (Synbiotic versus Control, *P* < 0.0001 and *P* = 0.005; Synbiotic versus Prebiotic, *P* < 0.0001 and *P* = 0.002, respectively). From the Spearman’s rank correlation coefficients analysis which was determined by combining the biological datasets from all 4 groups, we observed that increased abundance of Bifidobacteriaceae was positively correlated with fecal acetic acid and negatively correlated with stool pH (Fig. 5a). The increased abundance of strict anaerobes was positively correlated with acetic acid and conversely the increased abundance of facultative anaerobes and aerobes was negatively correlated with fecal levels of acetic acid (Fig. 5b). Additionally, an increased abundance of Lactic Acid Bacteria (LAB) [34] was positively correlated with fecal lactic acid and acetic acid (Fig. 5c). We hypothesized that Bifidobacteriaceae was the most abundant representative of the strict anaerobic and LAB functional taxa (Supplementary Fig. 3): when the relative abundance data of Bifidobacteriaceae was omitted from this comparison, the observed correlation coefficients became weaker and statistically non-significant. Similarly, we hypothesized that members of Enterobacteriaceae were the main constituents of the facultative anaerobic and aerobic, and non-LAB functional taxa: the positive correlation observed between abundance of facultative anaerobes/aerobes and stool pH became statistically non-significant when the variable Enterobacteriaceae was omitted from the former functional category.

### A synbiotic intervention prevents the establishment of a compromised microbiome in elective C-section born infants

Bacterial species abundances were determined by metagenomics analysis (Supplementary Fig. 4). The abundance of *Bifidobacterium breve* was positively correlated with differential abundance and expressed genes involved in milk carbohydrates (i.e. Lacto-N-I Galacto-N-Biose) metabolism and fatty acid biosynthesis indicating a fermentative metabolism in the Synbiotic group (Fig. 5d and Supplementary Figs. 5, 6, 7 and Tables S11, S12). Additionally, metabolomic analyses of pooled fecal samples revealed lower levels of lactose and HMO species in the Synbiotic and Reference groups compared to the Control and Prebiotic groups (Supplementary Fig. 8). The detection of HMOs in the fecal samples reflects the infants being mixed fed. Across these pooled samples, fecal lactose and HMOs had divergent abundance patterns compared to *Bifidobacterium,* while acetic acid and lactic acid had matching abundance patterns, thus suggesting a role for *Bifidobacterium* in metabolizing these carbohydrates and modulating the hypoxic and acidic milieu (Supplementary Fig. 9). Abundance of members of the genus *Klebsiella* within Enterobacteriaceae*,* of which *Klebsiella pneumoniae* was the dominant constituent, was positively correlated to differentially expressed genes involved in respiration (i.e. formate dehydrogenase), virulence, lipopolysaccharide (LPS) biosynthesis (i.e. KDO2-Lipid A) and stress response (reactive oxygen species metabolism: i.e. Glutathione S-transferase) (Fig. 5d and Supplementary Figs. 4, 5, 6, 10 and Tables S11, S12).

## Discussion

This study demonstrated that infants born by elective C-section present a microbiome characterised by meta-omics signatures of increased gut redox potential resulting from delayed establishment of strict anaerobes. In this study the reference group included vaginally born infants which were not exposed to intrapartum antibiotic prophylaxis (IAP). IAP and postnatal antibiotic administration are known factors that could potentially mask or alter the effects of mode of delivery [35–37]. Several studies describe the impact of IAP on the gut microbiome of infants born vaginally and have shown that IAP results in a delayed colonization by *Bifidobacterium* and enrichment of Enterobacteriaceae [36, 38, 39]. The elective C-section born infants in the intervention and control groups were exposed to IAP administration according to the standard clinical practice to prevent post-caesarean maternal infection. Previous studies indicated that infants born by elective C-section present a compromised microbiome (delayed colonization by *Bifidobacterium* and enrichment of Enterobacteriaceae) irrespective of IAP exposure. No differences were observed between those born by C-section with IAP or without IAP exposure [20, 21, 40].

We demonstrated that the synbiotic intervention modulated the anaerobic catabolism in the gut of infants born by elective C-section from the first days of life, emulating the microbial catabolism observed in vaginally born infants [41, 42]. The single probiotic strain within the synbiotic normalized the species diversity by restoring the *Bifidobacterium* genus levels without impeding the colonisation and expansion of endogenous *Bifidobacterium* species (Supplementary Fig. 11) and other bacterial taxa. *Bifidobacterium* appeared as the key driver in modulating anaerobiosis through the production of organic acids, and the resulting acidification of the intestinal milieu. We hypothesize that the presence of *Bifidobacterium* in the first days of life is paramount to the establishment of gut anaerobiosis. The transmission of *Bifidobacterium* during natural birth and breastfeeding highlights the role of the maternal microbiome in establishing *Bifidobacterium* as a paramount early colonizer of the infant gut. The presence of *Bifidobacterium* in maternal faeces, vagina and breast milk has been documented and supports its key biological role in establishing the anaerobiosis in early life [25, 43–46]. This vertical transfer concept was clearly illustrated by Makino and colleagues who demonstrated that mothers who gave birth vaginally transmitted their unique family-specific *Bifidobacterium* strains to their infant’s gut. *Bifidobacterium* strains from a mother who gave birth to twins were identified in both siblings [25].

Impaired acquisition of *Bifidobacterium* from the first days of life delays the establishment of anaerobiosis, and consequently the acidification of the gut milieu does not occur. The delayed colonization by *Bifidobacterium* sp. was correlated to a depletion of genes involved in milk carbohydrates metabolism. This impaired transmission of *Bifidobacterium* seemed to be reflected by an enrichment of HMOs and lactose in the stool samples of C-section born infants from the Control and Prebiotic groups even though they also received breast milk. Our study indicates that infants born with a compromised microbiome (delayed colonization by *Bifidobacterium*) would benefit from a nutritional supplementation that complements the *Bifidobacterium* driven utilization of milk carbohydrates present in breast milk. These insights were inferred using data coming from pooled samples; hence they could have been caused by one or more of the pooled samples. A recent study corroborated our findings and demonstrated that the presence of *Bifidobacterium* supplemented in breast milk fed preterm infants was associated with higher fecal acetic acid and lactic acid, lower stool pH and the consumption of HMOs [47].

Here, we demonstrated that the very first days of life form a time frame in which the impact of mode of delivery on the ecophysiology of the gut microbiome is most profound. In comparison, other studies investigated the development of the gut microbiome from the first month of life or 3 months onwards [6, 8, 48]. The lack of data collected during this window period led Bokulich and colleagues to argue that the first month of life is dominated by facultative aerobes [48], whereas this study has demonstrated that the establishment of strict anaerobes already occurs in the first days of life.

Furthermore, our findings indicate that birth by elective C-section is associated with a compromised microbiome enriched with facultative anaerobes and aerobes. Those early colonizers in C-section born infants are acquired from the surrounding birth environment (hospital environment). Opportunistic pathogens associated with the hospital environment, carrying antimicrobial resistance genes belonging to the Enterobacteriaceae or Enterococcaceae have been identified in the gut of infants born by C-section [27]. Additionally, dust from operating rooms collected right after C-section surgery, was found to contain human skin bacteria [49]. Those shed human skin bacteria might also contribute to seed the microbiome of infants born by C-section. A gut microbiome depleted of strict anaerobes and enriched for facultative anaerobes and aerobes is documented for (1) infants born vaginally whose mothers received IAP, (2) antibiotic treated infants, (3) preterm born infants, (4) malnourished children and (5) children afflicted with infectious diarrhoea [35, 36, 50–53]. These biological observations indicate that a compromised microbiome is often associated with an expansion of Enterobacteriaceae that favours an aerobic catabolism [54–56]. Recently, two studies demonstrated that antibiotic treatment resulted in depletion of fiber degrading bacteria (strict anaerobes) from the gut lumen; this microbial perturbation led to a depletion of short chain fatty acids (SCFA) that deranged the metabolism of the colonic epithelium [57, 58]. The luminal depletion of SCFA as a source of energy for the colonocytes prevented the latter from consuming oxygen through β-oxidation, leading to an increased epithelial oxygenation that drove the aerobic expansion of Enterobacteriaceae. Mariana X. Byndloss hypothesizes that “colonocyte metabolism plays a central role in balancing the gut microbiota by “suffocating harmful bacteria” [59]. In support, Million and colleagues described an increased redox potential associated with a depletion of strict anaerobic in the gut microbiome of malnourished children [50]. Our data suggest that *K. pneumoniae,* as the dominant representative of the *Klebsiella* genus, might be a key contributor in modulating the aerobiosis through respiration and reactive oxygen species metabolism. This metabolic activity was also associated with the differential expression of genes involved in LPS biosynthesis. The role of LPS in modulating immunity in early life has not been explored extensively [60]. Our finding indicates the need of investigating the effect of the release of LPS in the colonic environment of infants born by elective C-section.

Hence, we propose that the presence of *Bifidobacterium* in the first days of life could have a key biological significance in modulating the redox and acidity of the gut environment, which provides colonization resistance. *Bifidobacterium* uses a unique central fermentative pathway called the “bifid shunt” which equips bifidobacteria with an unique evolutionary advantage of generating more ATP (per mole of glucose) in comparison to microorganisms using other carbohydrate fermentative pathways such as glycolysis. We hypothesize that acetic acid, one of the bifid shunt’s end-products, is oxidized by colonocytes and contributes to augmentation of the epithelial barrier function. In support, Fukuda and colleagues demonstrated the role of acetate-producing *Bifidobacterium* in enhancing immunity to bacterial infection by acting on the colonic epithelium [61, 62]. The exclusion of oxygen from the gut in the first days of life is likely a crucial step in driving microbiome succession towards a resilient, stable, and healthy state [63]. The process of childbirth, the transition from the hypoxic womb environment to the hyperoxic extra-uterine environment, causes an oxidative stress challenge [64, 65]. This observation deserves to explore any biological link between the oxidative stress of birth and the establishment of *Bifidobacterium* in early life or the early life microbiome. Several studies have demonstrated that a depletion of *Bifidobacterium* and an enrichment of *Klebsiella* [66] in the first 100 days of life is a risk factor for the development of paediatric allergy [3, 5, 66], emphasizing the instrumental role of *Bifidobacterium* in programming the immune system in early life [5, 30, 67, 68]. The delayed colonization by *Bifidobacterium* is an early microbial signature that resolves several weeks after the C-section delivery [18]; however, little is known on the impact of this delayed establishment of microbes with the ability to consume HMOs on the development of the gut microbiome during infancy. Interestingly, insights from the Danish birth cohort identified a subgroup of C-section born infants who presented a reduction of microbiome members with the ability to digest or ferment dietary fibers into short chain fatty acids (SCFAs) at 1 year of age. Those infants were associated with an increased risk of asthma later in life. The authors hypothesize that infants born by C-section whose microbiome does not recover and mature properly in the first year of life have an increased risk to develop asthma [69].

In our study population, we confirmed the delayed colonization by *Bacteroide*s in C-section born infants [20, 41]. Little is known about the role of *Bacteroides* in vaginally born infants except their ability to metabolize HMOs, suggesting therefore a role in modulating the gut anaerobiosis. Their implication in early life deserves further attention [20, 26, 70] though two recent birth cohort studies suggest the role of *Bacteroides* in allergy prevention [71, 72].

## Conclusions

This study demonstrated that elective C-section birth results in a delayed establishment of hypoxia in the gut that favours the expansion of facultative anaerobes and aerobes, that do not have the genomic ability to metabolize milk carbohydrates. We hypothesize that this observed biological phenomenon could be described as a hallmark of a compromised microbiome, connected to the biosynthesis of LPS and the metabolism of reactive oxygen species in the colonic environment. This functional microbial hallmark might underline the role of a compromised microbiome as a risk factor for the development of non-communicable diseases. The role of LPS and oxidative stress as a reflection of reactive oxygen species metabolism in the context of early life microbiome deserve some scientific attention (Supplementary Figures 12 and 13). In vivo or ex vivo studies will be needed to test the hypotheses raised in our study.

Our study suggests that supplementation with a specific synbiotic microbiome modulator allows the establishment of an acidic gut ecosystem devoid of oxygen in infants with a compromised microbiome at birth. The long-term health consequences of this early life nutritional intervention have not been assessed, however children that participated in this study are currently being followed up.

## Methods

Our study adheres to the CONSORT guidelines, please refer the CONSORT checklist.

### Trial

This was an exploratory, randomized, double-bind, controlled study conducted between June 2011 and April 2013 in Singapore and Thailand. All participating centres obtained approval of their independent local Ethical Review Board (SingHealth Centralised Institutional Review Board in Singapore (reference number is CIRB Ref: 2010/832/E) and Institutional Review Board, Faculty of Medicine, Chulalongkorn University in Thailand (reference number is IRB No. 437/55)). Written informed consent was obtained from all parents before randomization. The study was registered in the Dutch Trial Register (http://www.trialregister.nl/NTR Number: 2838). All details of the clinical study such as intervention exposure can be found in the clinical trial publication. Subjects from all the groups were mixed fed, they received the study product corresponding to their allocated group in addition to breastfeeding. Additionally, the majority of the subjects in the Reference group was mixed fed and received in addition to human milk the Control product (23/30 subjects; 77%) [18]. All the infants born by elective C-section were exposed to intrapartum antibiotic prophylaxis (IAP) to prevent post-caesarean maternal infection. Among those born vaginally from the reference group (*n* = 30), two infants were exposed to IAP and were excluded from this study. Here, we leveraged the fecal samples collected in this clinical trial and deployed a multi-omics approach (16S rRNA amplicon sequencing, shotgun metagenomics, metatranscriptomics and metabolomics) to deconvolute the eco-physiological impact of mode of delivery and nutrition on the early-life gut microbiome (Fig. 1).

### Microbiome multi-omics data

All the biological data (16S rRNA, SCFA, Lactate and pH, metagenomics, metatranscriptomics and metabolomics) were generated from the modified intention-to-treat (mITT) population. The mITT population consisted of all randomized subjects who provided at least 1 baseline and post-baseline stool sample. In this study, we could only include those subjects whose fecal samples were of sufficient volume and quality for DNA extraction and 16S rRNA sequencing. However, this accounted for the vast majority of the subjects from mITT [18]. In this study, the number of subjects per group whose faecal genomic DNA was available for 16S rRNA sequencing, was as follows: Synbiotic group (*n* = 44), Prebiotic group (*n* = 39), Control group (*n* = 44) and Reference group (*n* = 26) (Supplementary Table S1). For data analysis and interpretation, these intervention groups were labelled as Synbiotic group, Prebiotic group, Control group and Reference group. In total, bacterial community compositions were characterised by shotgun 16S rRNA sequencing of 943 faecal genomic DNA samples (Supplementary Table S1). The metagenomics and metatranscriptomics data were generated from a subset of the stool samples collected at day 3 and/or day 5 (Fig. 1). The samples were selected randomly from 10 subjects per group from the clinical study’s biobank. The metabolomics data were generated from a subset of the stool samples collected at day 3 and/or day 5 (Fig. 1). The samples were selected randomly from 10 subjects per group from the clinical study’s biobank. The 10 samples per group were then pooled before metabolomics profiling. The metabolomics dataset represented four pools reflecting the intervention and reference groups.

### Multi-omics methods

#### Shotgun 16S rRNA sequencing of the V3-V6 region

##### Nucleic acid extraction, 16S rRNA amplification and DNA sequencing

Faecal genomic DNA was extracted using a combination of mechanical and chemical lysis via FastPrep Instrument (MP Biomedicals) and QIAamp Fast DNA Stool Mini Kit (Qiagen) as described previously [18]. The V3-V6 region on prokaryotic 16S rRNA was amplified from total DNA as previously described [73–76]. Amplicons were sheared using the Covaris LE220 sonicator (Covaris, Inc., USA) and built into sequencing libraries using GeneRead DNA Library I Core Kit (Qiagen) according to the manufacturer’s protocol. DNA libraries were multiplexed by 96 indices, pooled, and sequenced on the Illumina HiSeq 2500 using paired-end (2x76bp) sequencing. Sequencing reads were demultiplexed (Illumina bcl2fastq 2.17.1.14 software) and filtered (PF = 0) before conversion to FASTQ format.

##### Sequencing output

The average numbers of sequencing quality-passed reads mapping to the 16S rRNA Greengenes global rRNA database (dated May 2013; greengenes/13_5/99_otus.fasta) are described in the Supplementary Table S2.

##### Reconstruction and classification of 16S rRNA amplicon sequences

Read trimming from 3′ was done to remove bases with quality score ≤ 2, followed by additional removal of reads pairs shorter than 60 bp after trimming [73–77]. Following read trimming, full length 16S rRNA (V3-V6 region) reconstructions were produced from the short sequencing reads using the EMIRGE amplicon (Expectation Maximisation Iterative Reconstruction of Genes from the Environment) algorithm [75–78]. The analysis method is described in the publication of Ong and colleagues [73] and the updated tools and pipeline are available at: https://github.com/CSB5/GERMS_16S_pipeline. EMIRGE leverages 16S rRNA sequences on the SILVA database for template guided assembly of reconstructions of the 16S rRNA amplicon sequences [77, 78]. EMIRGE prevents chimeric sequences from mapping. The reconstructed 16S rRNA sequences (at least 99% sequence similarity) are collapsed into OTUs, and Graphmap [79] was used to map these OTUs to the Greengenes global rRNA database (dated May, 2013; greengenes/13_5/99_otus.fasta) [80]. OTUs were called at various taxonomic levels of identity (species, genus, family etc.) [81]. EMIRGE assigns abundance estimates to the reconstructed 16S rRNA sequences. The relative abundance of OTUs was determined for each sample and converted to relative abundances at various taxonomic level (phylum, family, genus, species level).

#### Metagenomics and metatranscriptomics

##### Nucleic acid extractions

DNA and RNA extractions were carried out with the ZR Fecal DNA MicroPrep and ZR Fecal RNA MicroPrep kits (Zymo Research), respectively, following the manufacturer’s protocols that are supplied with the kits. The homogenization step was performed on a FastPrep-24 instrument (MP Biomedicals). For each extraction, ~ 50-100 mg of feces were used. For RNA extractions, the optional DNase digestion step was performed to eliminate traces of contaminating DNA. Extracted nucleic acids were quantitated with Invitrogen’s Picogreen (DNA) and Ribogreen (RNA) assays prior to next generation sequencing library preparation.

##### Library preparation & next-generation sequencing

RNA library preparation was performed according to Illumina’s TruSeq Stranded mRNA protocol with the following modifications: The oligo-dT mRNA purification step was omitted and instead, 200 ng of total RNA were directly added to the Elution2-Frag-Prime step. The PCR amplification step, which selectively enriches for library fragments that have adapters ligated on both ends, was performed according to the manufacturer’s recommendation but the number of amplification cycles was reduced to 12. All libraries were dual barcoded with Illumina’s TruSeq HT RNA barcodes to enable library pooling for sequencing.

DNA library preparation was performed according to Illumina’s TruSeq Nano DNA Sample Preparation protocol. The samples were sheared on a Covaris S220 or E220 to ~ 450 bp, following the manufacturer’s recommendation. All libraries were dual barcoded with Illumina’s TruSeq HT DNA barcodes to enable library pooling for sequencing.

Finished libraries were quantitated using Invitrogen’s Picogreen assay and the average library size was determined on a Bioanalyzer 2100, using a DNA 7500 chip (Agilent). Library concentrations were then normalized to 4 nM and validated by qPCR on a ViiA-7 real-time thermocycler (Applied Biosystems), using the Kapa library quantification kit for Illumina platforms (Kapa Biosystems). DNA libraries were then pooled at equimolar concentrations and sequenced on an Illumina HiSeq2500 sequencer in rapid mode at a read-length of 250 bp paired-end. RNA libraries were also pooled at equimolar concentrations and sequenced on an Illumina HiSeq2500 sequencer in rapid mode at a read-length of 100 bp paired-end.

##### Sequencing data analysis

The raw metagenomics and metatranscriptomics Illumina reads were adapter-trimmed and quality-trimmed using cutadapt-1.8.1 [82] with parameter of “*-q 20 --trim-n --minimum-length 30 --match-read-wildcards*”. Afterwards, for each paired-end read dataset, the first pair and its corresponding second pair were locally assembled/merged using FLASH 1.2.6 [83] with *“-m 10 -M 251 -x 0.25*” and *“-m 10 -M 101 -x 0.25*” parameter for metagenomics and metatranscriptomics dataset respectively.

The metagenomics dataset was mapped against the hg19 human reference genome with bowtie2 2.2.5 [3] using “--*very-sensitive-local*” preset as its sensitivity parameter. Any reads that can’t be confidently mapped against the human genome, which were generated by “*--un*” switch, were separated and processed as the non-host reads. The non-host reads were then aligned against NCBI non-redundant protein database (downloaded on 3 January 2016) using Diamond version 0.85 [84] with default parameters. Based on these alignments, the microbial taxonomical classification was determined using the Lowest-Common-Ancestor (LCA) algorithm implemented in MEGAN6 [85] (parameters: *maxmatches = 25 minscore = 100 minsupport = 25*). The subsequent taxonomy and KEGG/SEED functional annotation were done as part of MEGAN6 processing and was based on MEGAN’s *gi2tax-July2016.bin* and *gi2keggMarch2016.bin* database files.

The metatranscriptomics dataset was mapped against hg19 human reference genome with bowtie2 2.2.5 [86] using “--*very-sensitive-local*” preset as its sensitivity parameter. The non-human reads were then separated into its ribosomal RNA and non-ribosomal RNA components using sortMeRNA 2.1 [87]. The non-ribosomal reads were then aligned against NCBI non-redundant protein database (downloaded on 3 January 2016) using Diamond version 0.85 [4] with default parameters. Based on these alignments, the microbial taxonomical classification was determined using the Lowest-Common-Ancestor (LCA) algorithm implemented in MEGAN6 [5] (parameters: *maxmatches = 25 minscore = 80 minsupport = 25*). The subsequent taxonomy and KEGG/SEED functional annotation were done as part of MEGAN6 processing and was based on MEGAN’s *gi2tax-July2016.bin* and *gi2keggMarch2016.bin* database files.

The metagenomics sequencing run produced, on average, 8.6 million paired-end reads per sample, or 4.3Gb data per-sample. After quality control and removal of host sequences, on average, 6,864,061 (79.7%) paired-end reads were used for downstream metagenomics analysis. At the end of the classification analysis, on average, 88.93% (66.78–94.97%) of the paired-end reads could be classified to the kingdom Bacteria.

The metatranscriptomics sequencing run produced, on average, 11.9 million paired-end reads per sample, or 2.4Gb data per-sample. After quality control and removal of ribosomal RNA sequences, on average, 1,212,609 (10.12%) paired-end reads were categorized as non-ribosomal RNA reads and used for downstream metatranscriptomics analysis. The resulting analysis shows that, on average, 60.31% (40–75.28%) of the non-ribosomal RNA reads could be classified to the kingdom Bacteria.

### *Targeted fecal metabolites and pH measurements*

Sample preparation, SCFA, lactate and pH measurement are described in the publication of Knol and colleagues [88].

Acetic acid was the main SCFA detected in the clinical samples as described in the clinical trial publication [18]. Acetic acid, lactic acid and stool pH data (stool physiological parameters) were correlated with the 16S rRNA data as described in the statistical analysis section.

#### Metabolite profiling study on pooled stool samples

##### Sample preparation

Individual stool samples were first lyophilized under vacuum using a freeze dryer (Christ Alpha) and an equal amount of dry mass weight for each sample was combined to prepare the pooled sample for each group. 40 mg of each pooled sample was added to 80 mg of glass homogenisation beads (acid washed, 425-600 μm, Sigma-Aldrich, USA) and processed using a two-phase modified Bligh and Dyer extraction protocol (Fukawa et al., 2016). Briefly, polar and lipid metabolites were extracted by sequential addition of methanol (Optima grade, Fisher Scientific), 3.8 mM tricine solution (Sigma-Aldrich) and chloroform (Gradient grade, Merck) (1:0.5:1 v/v/v, total 2 mL) to the sample. The mixture was vortexed for 2 min following each addition of solvent. The mixture was then centrifuged at 4 °C, 16,000 g for 20 min. This resulted in separation of the sample into two fractions – the top methanol-tricine solution layer contained the polar metabolites while the bottom chloroform layer contained the lipid species. The top polar layer was collected and the remaining mixture was re-extracted using a mixture of methanol and tricine solution (9:10 v/v), followed by centrifugation at 4 °C, 16,000 g for another 10 min. The resulting polar layer was combined together with the first polar extract and stored at -80 °C before subsequent mass spectrometry analysis.

##### Liquid chromatography and mass spectrometry (LC-MS)

Each polar extract was analysed in triplicate using an ultra-performance liquid chromatography system (AQUITY UPLC, Waters) in tandem with a mass spectrometer (QExactive, Thermo Scientific). A C18 UPLC column (ACQUITY UPLC HSS T3 column, 2.1 × 100 mm, 1.8 μm, Waters) was used for separation and the mobile phase comprised of two solvents. ‘A’ being water with 0.1% formic acid (Merck) and ‘B’ being methanol with 0.1% formic acid. The UPLC program is as follows: the column was first equilibrated for 0.5 min at 0.1% B. The gradient was then increased from 0.1% B to 50% B over 8 min before being held at 98% B for 3 min. The column was washed for a further 3 min with 98% acetonitrile (Merck) with 0.1% formic acid and finally equilibrated with 0.1% B for 1.5 min. The solvent flow rate was set at 0.4 mL/min; a column temperature of 30 °C was used. The eluent from the UPLC system was directed into the MS.

High-resolution mass spectrometry was then performed in both positive and negative electrospray ionization (ESI) modes, with a mass range of 70 to 1050 m/z and a resolution of 70,000. Sheath and auxiliary gas flow was set at 30.0 and 20.0 (arbitrary units) respectively, with a capillary temperature of 400 °C. The spray voltages were 1.25 kV for positive and 1.5 kV negative mode ionization. Mass calibration was performed using standard calibration solution (for QExactive, Thermo) prior to injection of the samples. A quality control (QC) sample comprising of equal aliquots of each sample was run at regular intervals during the batch LC-MS runs.

##### LC-MS data processing and analysis

The raw LC-MS data obtained was processed using a XCMS-based peak finding algorithm [89]. The QC samples were used to adjust for instrumental drift. Detected mass peaks were assigned putative metabolite identities by matching the respective masses (< 10 ppm error) with the KEGG and Human Metabolome Database (HMDB). Where possible, the identities of selected metabolites of interest were confirmed based on mass spectral comparison with available metabolite standards.

### *Statistical analysis*

Differences in bacterial community composition as determined by 16S rRNA sequencing among the four infant groups were assessed using distance-based redundancy analysis (db-RDA). The dissimilarity data based on Bray-Curtis distances were first ordinated using metric scaling and the results were then analysed using redundancy analysis. These steps were implemented using the capscale function from the vegan package in R software version 3.4.1 [90]. These analyses were carried out for each time point.

Permutational Multivariate Analysis of Variance (PERMANOVA) was applied on the ordination object to assess if microbial community composition differences were statistically significant between every infant group pairs. The envfit function of the vegan package was used to fit the vectors representing different bacteria onto the ordination object (999 permutations). This allowed to investigate and visualize relationships among the different bacteria groups influencing the overall microbial community structure in the dimensional space. The steps depicted above were applied to the family and genus datasets.

Prior to assess differences in relative abundances of bacterial taxa between study groups, bacterial taxa with an average relative abundance < 0.5% were removed. Non-parametric Mann-Whitney U tests were then performed at each time point on each pair of study group combination at family, genus and species level. Pairwise Wilcoxon test function from the R Stats package was used for implementation. For all statistical tests performed, adjusted Bonferroni *p*-values were derived to correct for multiplicity.

The Shannon index was calculated to assess alpha diversity for each sample (using the diversity function from vegan R package). The Spearman’s rank correlation coefficients between bacterial groups and stool physiological parameters were calculated together with the associated significance values. Based on a significance level of 0.05, Spearman’s rank correlation coefficients were filtered and exported to GraphPad Prism 7 for graphical representation.

The metagenomics and metatranscriptomics datasets (40 samples) were considered as one group regardless of the intervention group to determine Pearson correlations between key species and functional genes. All statistical analysis performed on the metagenomics and metatranscriptomics data were done with R statistical package version 3.4.0 using relative abundance data derived from MEGAN6 classification results. The metabolomics dataset represented four pools reflecting the intervention and reference groups, with each pool consisting of 10 random samples biologically pooled before metabolomics profiling. Organic acids, stool pH and 16S rRNA data were similarly grouped by the intervention and reference groups; differences between group means were compared descriptively.

## Supplementary Information

**Additional file 1: Figure S1.** Prevalence of *Bifidobacterium* and *Bacteroides* per intervention group from day 3 till week 22. **Figure S2a.** Box-plots representing the distribution of samples along db-RDA1 at week 2. Non-parametric Mann-Whitney U tests *P* < 0.05, *P* < 0.01 and *P* < 0.001 are indicated by *, ** and ***, respectively. **Figure S2b**. Box-plots representing the distribution of samples along db-RDA1 at week 4. Non-parametric Mann-Whitney U tests *P* < 0.05, P < 0.01 and P < 0.001 are indicated by *, ** and ***, respectively. **Figure S3.** Relative abundance of Lactic Acid Bacteria (LAB). Stacked bar plot of the relative abundance of Lactic Acid Bacteria from day 3 till week 22. C: Control, P: Prebiotics, R: Reference, S: Synbiotic. **Figure S4.** Top 10 bacterial species identified among the 40 metagenomics samples from the four groups. **Figure S5.** Metabolic pathway derived from the metagenomics dataset depicting correlations with *Bifidobacterium breve* (blue) and *Klebsiella* (red). KEGG genes which exhibit Pearson correlation coefficients > 0.6 against the metagenomics reads assigned to the two species are mapped onto the KEGG metabolism pathway map. **Figure S6.** Metabolic pathway derived from the metatranscriptomics dataset depicting correlations with *Bifidobacterium breve* (blue) and *Klebsiella* (red). KEGG genes which exhibit Pearson correlation coefficients > 0.6 against the metatranscriptomic reads assigned to the two species are mapped onto the KEGG metabolism pathway map. **Figure S7.** Correlation between *Bifidobacterium breve* and HMOs metabolism. Linear model plot depicting correlation between *Bifidobacterium breve* and Lacto-N I Galacto-N-biose metabolism (SEED data). **Figure S8.** Heat map of lactose and HMO species present in the pooled samples for the synbiotic, prebiotics, control and reference groups (in triplicate). The colour scale is based on the row z-score. **Figure S9.** Abundance patterns of *Bifidobacterium* (16S rRNA), milk sugars, acetate and lactic acid as emerged from biological pools and corresponding groups. *Bifidobacterium* and acetic acid were detected at their highest levels of abundance in correspondence to lactose and HMOs being detected at their lowest, and vice-versa. Raw intensities (for milk sugars), amounts (for organic acid) and relative abundances (for *Bifidobacterium*) were normalized between 0 and 1 to allow for a visual comparison. **Figure S10a.** Correlation between *Klebsiella* and respiration. Linear model plot depicting correlation between *Klebsiella* and Formate deshydrogenase (SEED data). **Figure S10b.** Correlation between *Klebsiella* and reactive oxygen species metabolism. Linear model plot depicting correlation between *Klebsiella* and Glutathion-S-transferase (SEED data). **Figure S10c.** Correlation between *Klebsiella* and lipopolysaccharide biosynthesis (LPS). Linear model plot depicting correlation between *Klebsiella* and KDO2-Lipid A biosynthesis (SEED data). **Figure S11.** Prevalence of human infant type *Bifidobacterium* species per group from day 3 till week 22. **Figure S12.** Infographic depicting the functional impact of vaginal birth on the infant gut microbiome. The acquisition of *Bifidobacterium* during birth allows the establishment of a hypoxic and acidic gut environment that prevents the growth of opportunistic pathogens (Enterobacteriacae). *Bifidobacterium* metabolizes the human milk oligosaccharides present in human milk through a fermentative metabolism, and acetate, one of the major end-products of the pathway, is oxidized by the colonocytes (β oxidation) as a source of energy. This biological phenomenon prevents the increase in oxygenation in colonic epithelial cells and contributes towards reducing and acidifying the colonic environment. **Figure S13.** Infographic depicting the functional impact of C-section birth on the infant gut microbiome. C-section birth is characterized by an enrichment of facultative anaerobes and aerobes (Enterobacteriaceae). The delayed colonization by *Bifidobacterium* prevents the establishment of a reduced and acidic gut environment leading to an increase in oxygenation in colonic epithelial cells and expansion of Enterobacteriaceae. Members of Enterobacteriaceae such as *Klebsiella pneumoniae*, uses oxygen to thrive in the colonic environment. They express lipopolysaccharide (LPS) and metabolize reactive oxygen species in the colonic environment.

**Additional file 2: Supplementary Table 1.** Number of subjects per group and number of 16S rRNA samples per group and time point.

**Additional file 3: Supplementary Table 2.** 16S rRNA sequencing outputs.

**Additional file 4: Supplementary Table 3.** Permutational Multivariate Analysis of Variance (PERMANOVA) tests.

**Additional file 5: Supplementary Table 4.** 16S rRNA Family data (Mean and SD) per group and time point.

**Additional file 6: Supplementary Table 5.** 16S rRNA Genus data (Mean and SD) per group and time point.

**Additional file 7: Supplementary Table 6.** 16S rRNA Species data (Mean and SD) per group and time point.

**Additional file 8: Supplementary Table 7.** Non-parametric Mann-Whitney U tests performed at each time point on each pair of study group combination at family level.

**Additional file 9: Supplementary Table 8.** Non-parametric Mann-Whitney U tests performed at each time point on each pair of study group combination at genus level.

**Additional file 10: Supplementary Table 9.** Non-parametric Mann-Whitney U tests performed at each time point on each pair of study group combination at species level.

**Additional file 11: Supplementary Table 10.** Categorization of bacterial families into functional groups (anaerobic/aerobic metabolism and Lactic Acid Bacteria).

**Additional file 12: Supplementary Table 11.** KEGG functional annotation.

**Additional file 13: Supplementary Table 12.** SEED functional annotation.

## Data Availability

Some of the datasets used and/or analysed during the current study are available in the supplementary information documents, additional datasets are available from the corresponding author on reasonable request. Raw 16S rRNA data are available at https://www.ebi.ac.uk, accession number is PRJEB44790. Shotgun Metagenomics and Metatranscriptomics data are available athttps://www.ncbi.nlm.nih.gov, accession number is PRJNA726032.

## Abbreviations

SCFA: Short chain fatty acids
IAP: Intrapartum antibiotic prophylaxis
LAB: Lactic acid producing bacteria
HMOs: Human milk oligosaccharides
LPS: Lipopolysaccharides
ATP: Adenosine triphosphate
