# Supplementary material for: A synbiotic intervention modulates meta-omics signatures of gut redox potential and acidity in elective caesarean born infants
Source: BMC Microbiol. 2021 Jun 25;21:191. doi: 10.1186/s12866-021-02230-1 (PMC8229302; doi:10.1186/s12866-021-02230-1)

**Supplementary figures**

Figure S1. Prevalence of *Bifidobacterium* and *Bacteroides* per intervention group from day 3 till week 22.

Figure S2a. Box-plots representing the distribution of samples along db-RDA1 at week 2. Non-parametric Mann-Whitney U tests P<0.05, P<0.01 and P<0.001 are indicated by *, ** and ***, respectively.

Figure S2b. Box-plots representing the distribution of samples along db-RDA1 at week 4. Non-parametric Mann-Whitney U tests P<0.05, P<0.01 and P<0.001 are indicated by *, ** and ***, respectively.

Figure S3. Relative abundance of Lactic Acid Bacteria (LAB).

Stacked bar plot of the relative abundance of Lactic Acid Bacteria from day 3 till week 22. C: Control, P: Prebiotics, R: Reference, S: Synbiotic

Figure S4. Top 10 bacterial species identified among the 40 metagenomics samples from the four groups.

Figure S5. Metabolic pathway derived from the metagenomics dataset depicting correlations with *Bifidobacterium breve* (blue) and *Klebsiella* (red).

KEGG genes which exhibit Pearson correlation coefficients > 0.6 against the metagenomics reads assigned to the two species are mapped onto the KEGG metabolism pathway map.

Figure S6. Metabolic pathway derived from the metatranscriptomics dataset depicting correlations with *Bifidobacterium breve* (blue) and *Klebsiella* (red).

KEGG genes which exhibit Pearson correlation coefficients > 0.6 against the metatranscriptomic reads assigned to the two species are mapped onto the KEGG metabolism pathway map.

Figure S7. Correlation between *Bifidobacterium breve* and HMOS metabolism.

Linear model plot depicting correlation between *Bifidobacterium breve* and Lacto-N I Galacto-N-biose metabolism (SEED data).

Figure S8. Heat map of lactose and HMO species present in the pooled samples for the synbiotic, prebiotic, control and reference groups (in triplicate).

The colour scale is based on the row z-score.

Figure S9. Abundance patterns of *Bifidobacterium* (16S rRNA), milk sugars, acetate and lactic acid as emerged from biological pools and corresponding groups.

*Bifidobacterium* and acetic acid were detected at their highest levels of abundance in correspondence to lactose and HMOs being detected at their lowest, and vice-versa. Raw intensities (for milk sugars), amounts (for organic acid) and relative abundances (for *Bifidobacterium*) were normalized between 0 and 1 to allow for a visual comparison.

Figure S10a. Correlation between *Klebsiella* and respiration.

Linear model plot depicting correlation between *Klebsiella* and Formate deshydrogenase (SEED data).

Figure S10b. Correlation between *Klebsiella* and reactive oxygen species metabolism.

Linear model plot depicting correlation between *Klebsiella* and Glutathion-S-transferase (SEED data).

Figure S10c. Correlation between *Klebsiella* and lipopolysaccharide biosynthesis (LPS).

Linear model plot depicting correlation between *Klebsiella* and KDO2-Lipid A biosynthesis (SEED data).

Figure S11. Prevalence of human infant type *Bifidobacterium* species per group from day 3 till week 22.

Figure S12. Infographic depicting the functional impact of vaginal birth on the infant gut microbiome.

The acquisition of *Bifidobacterium* during birth allows the establishment of a hypoxic and acidic gut environment that prevents the growth of opportunistic pathogens (Enterobacteriacae). *Bifidobacterium* metabolizes the human milk oligosaccharides present in human milk through a fermentative metabolism, and acetate, one of the major end-products of the pathway, is oxidized by the colonocytes (β oxidation) as a source of energy. This biological phenomenon prevents the increase in oxygenation in colonic epithelial cells and contributes towards reducing and acidifying the colonic environment.

Figure S13. Infographic depicting the functional impact of C-section birth on the infant gut microbiome.

C-section birth is characterized by an enrichment of facultative anaerobes and aerobes (Enterobacteriaceae). The delayed colonization by *Bifidobacterium* prevents the establishment of a reduced and acidic gut environment leading to an increase in oxygenation in colonic epithelial cells and expansion of Enterobacteriaceae. Members of Enterobacteriaceae such as *Klebsiella* *pneumoniae*, uses oxygen to thrive in the colonic environment. They express lipopolysaccharide (LPS) and metabolize reactive oxygen species in the colonic environment.


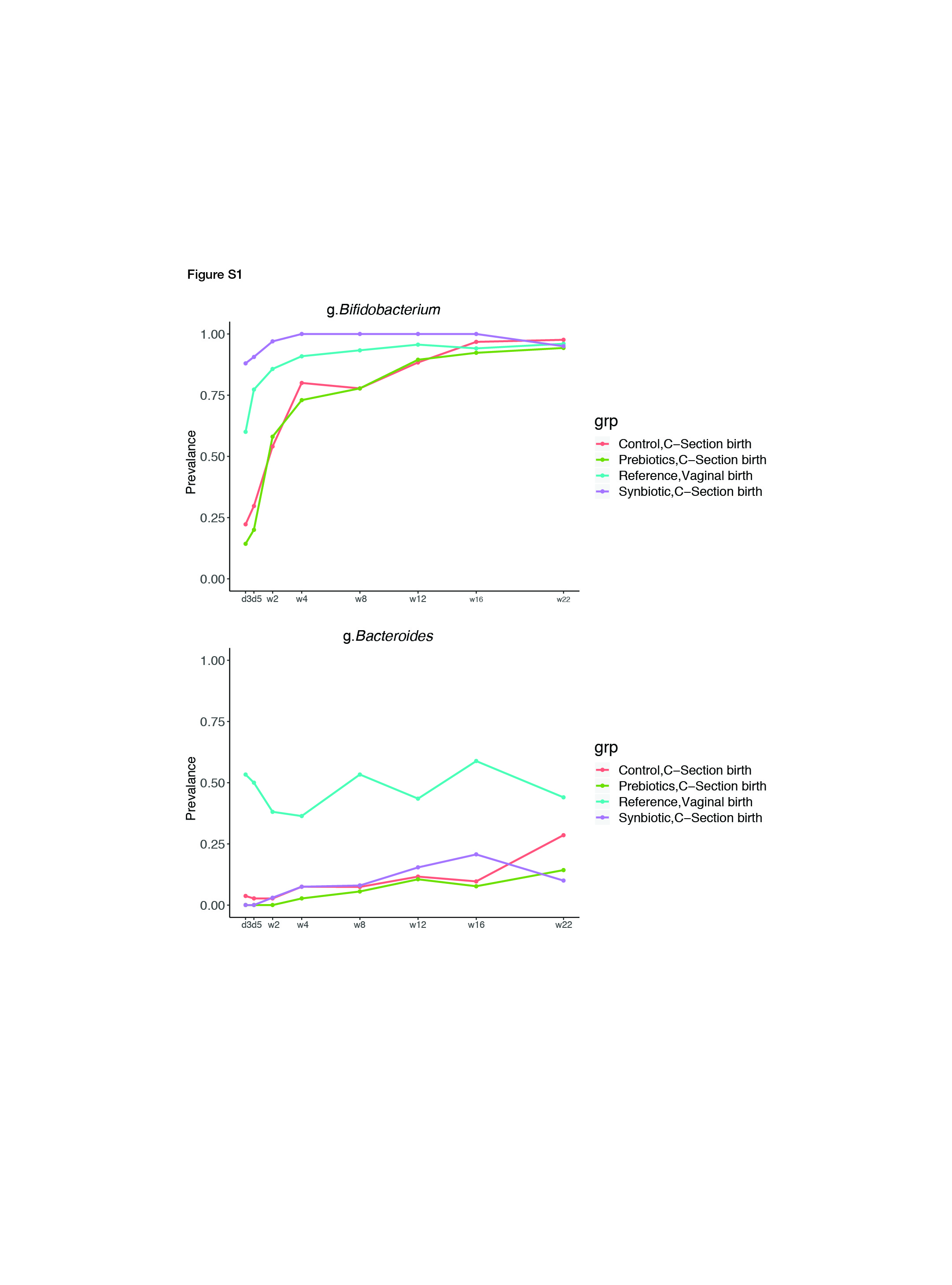


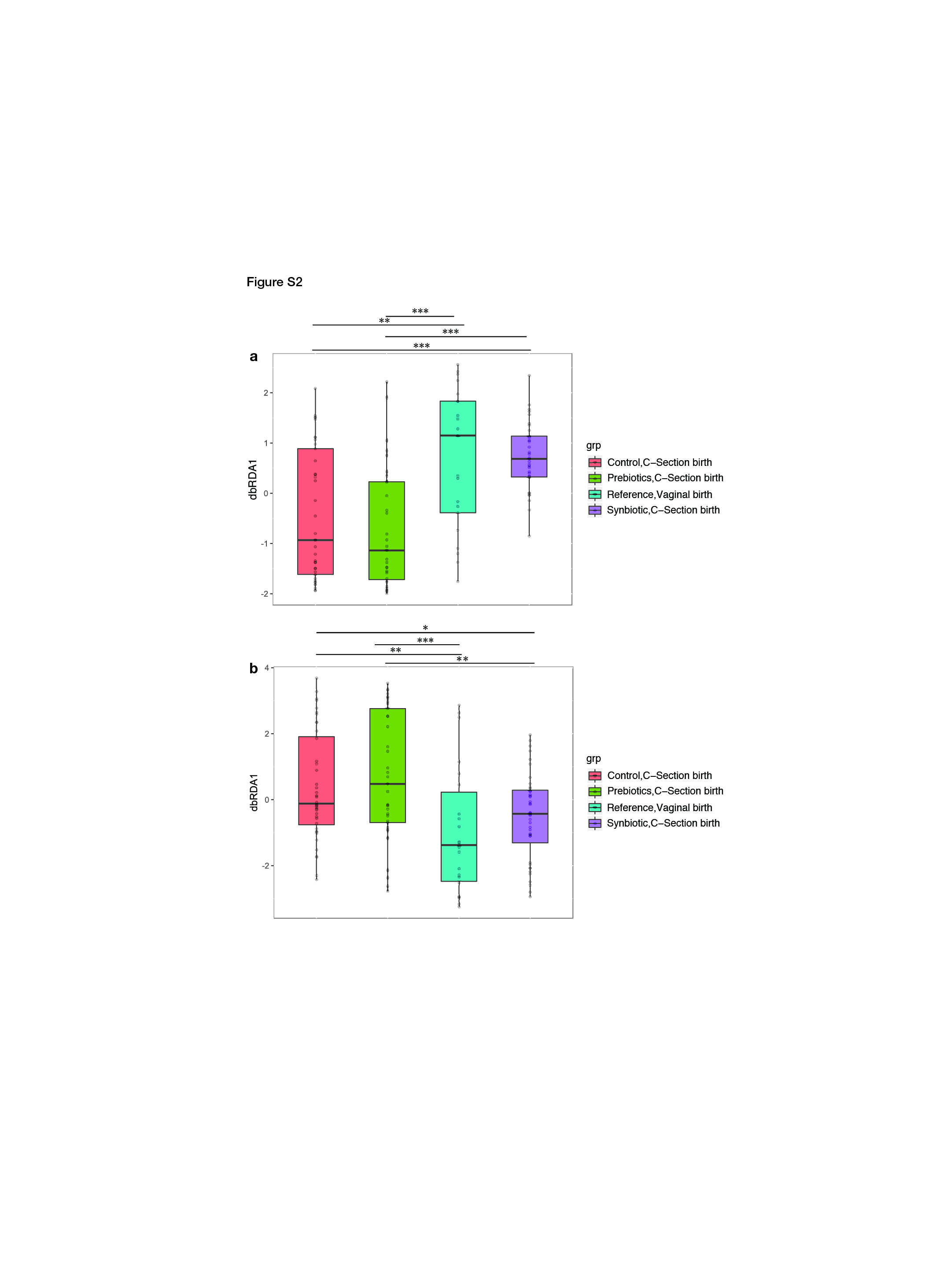


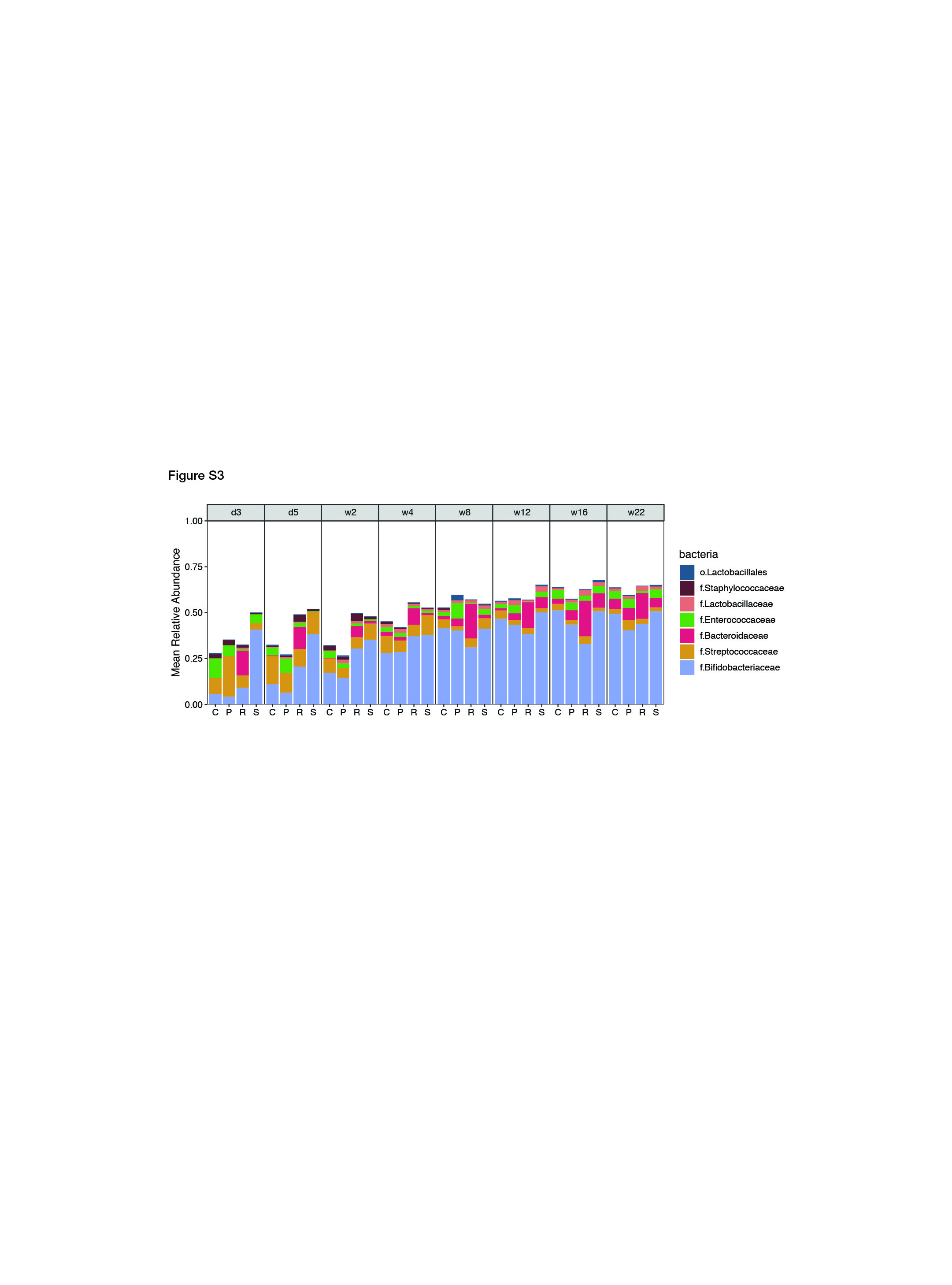


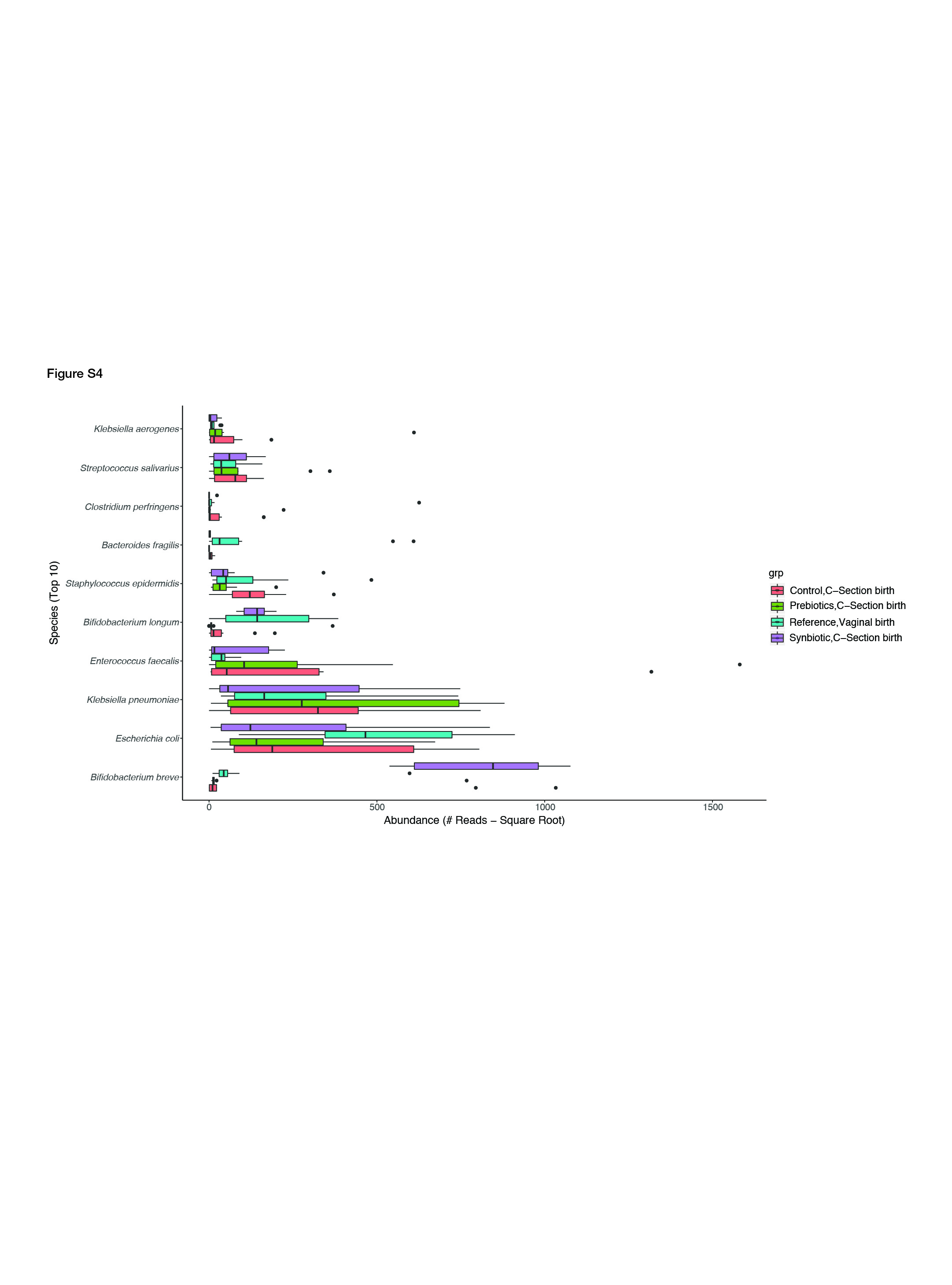


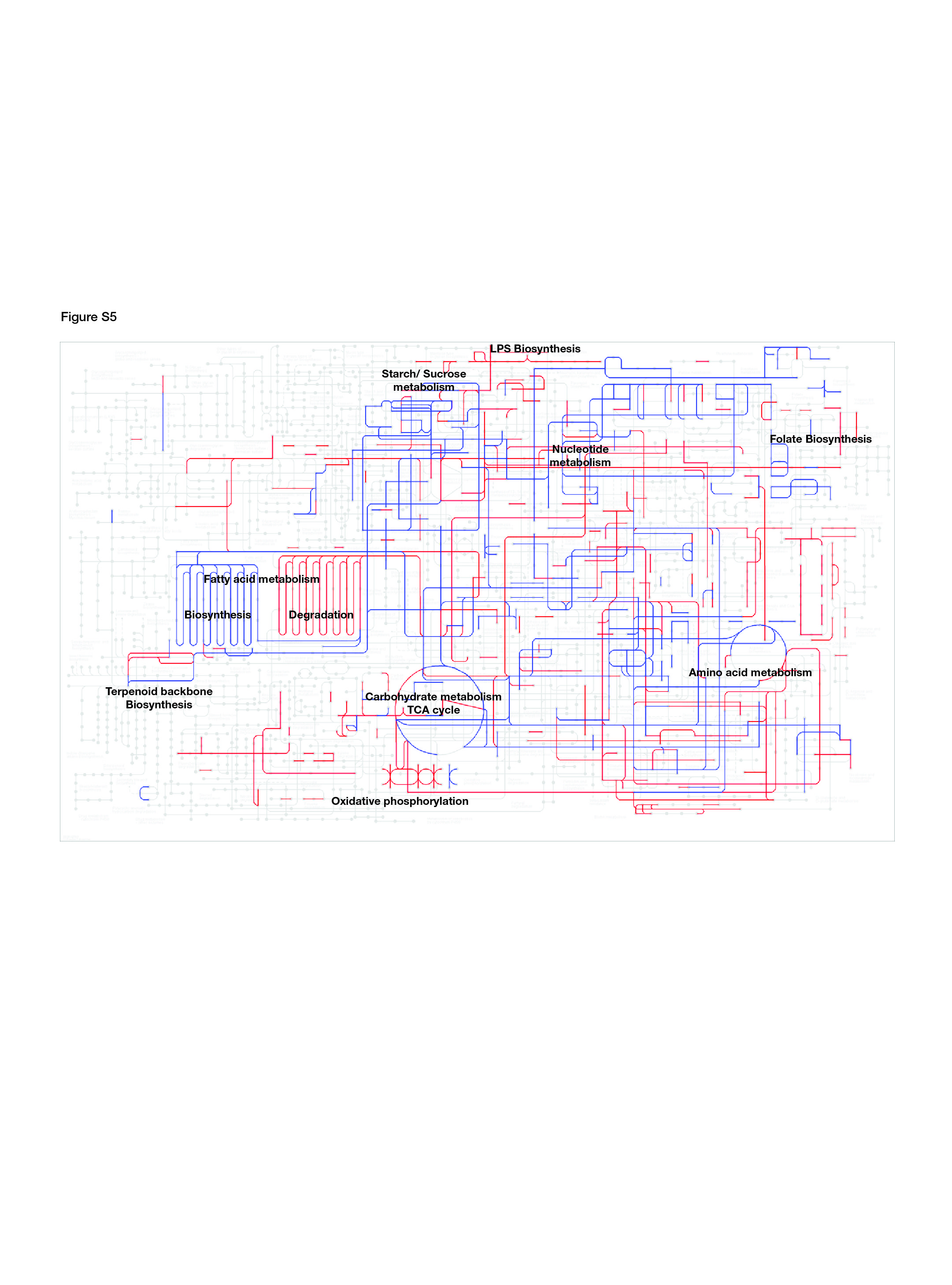


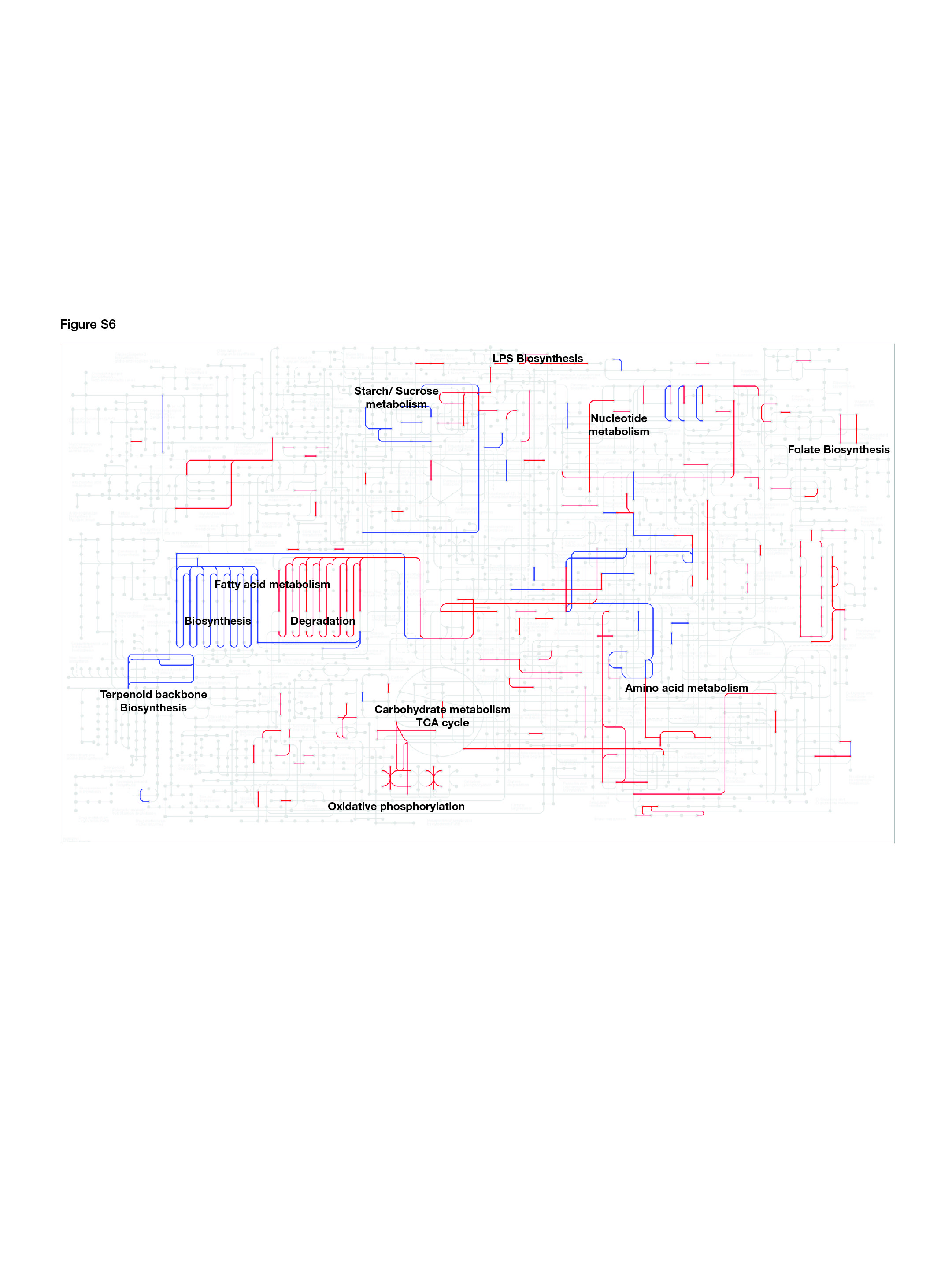


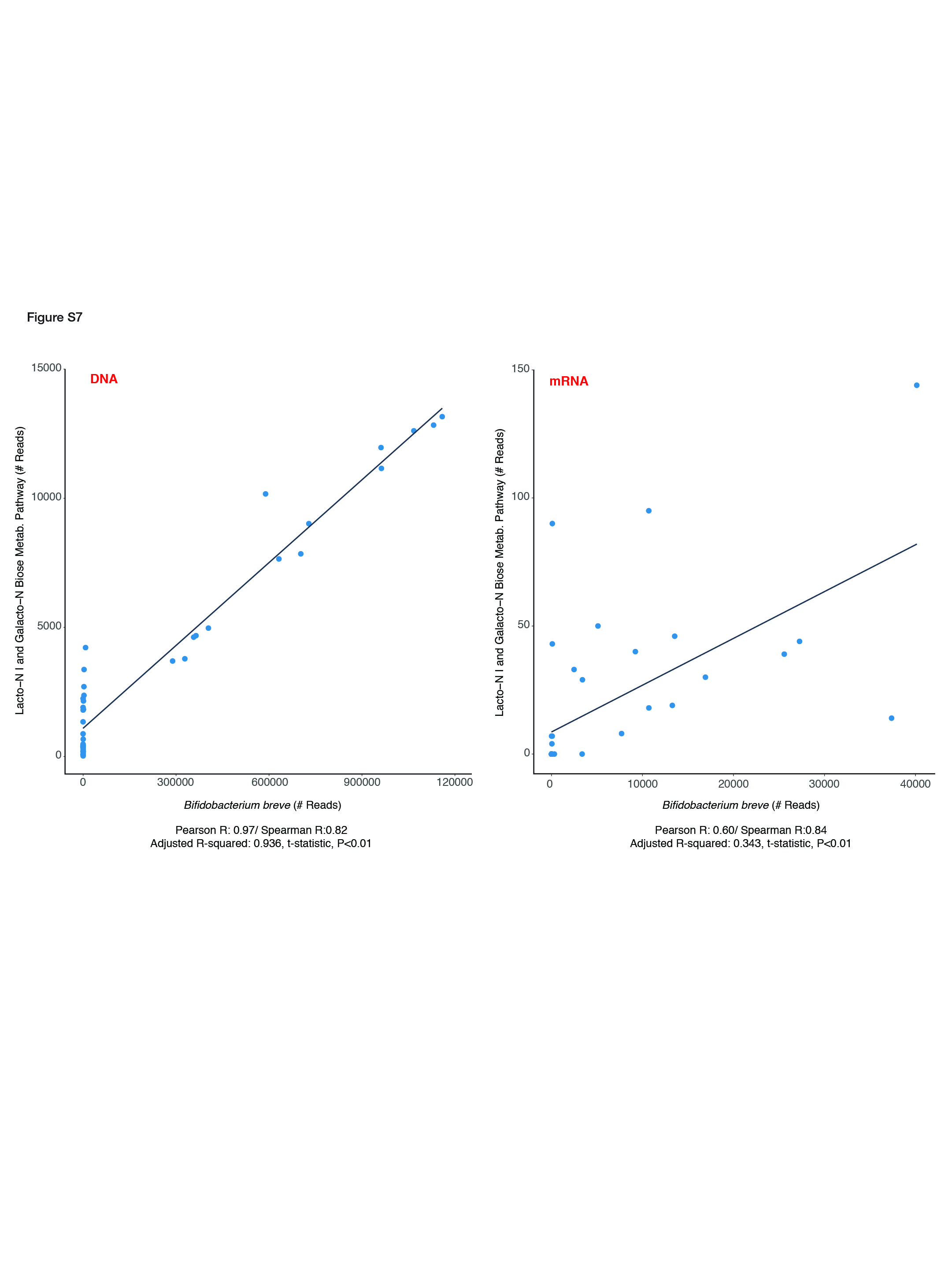


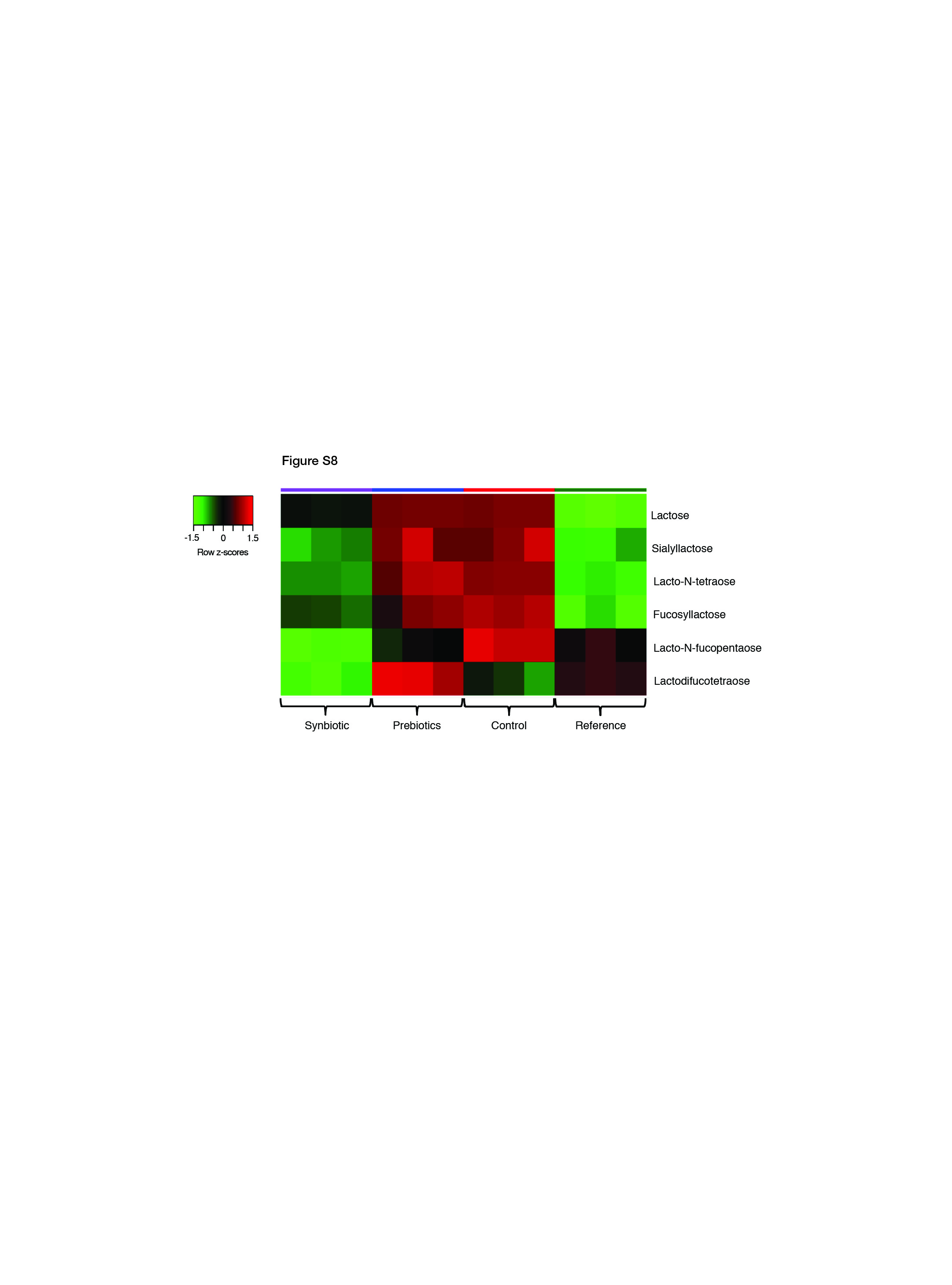


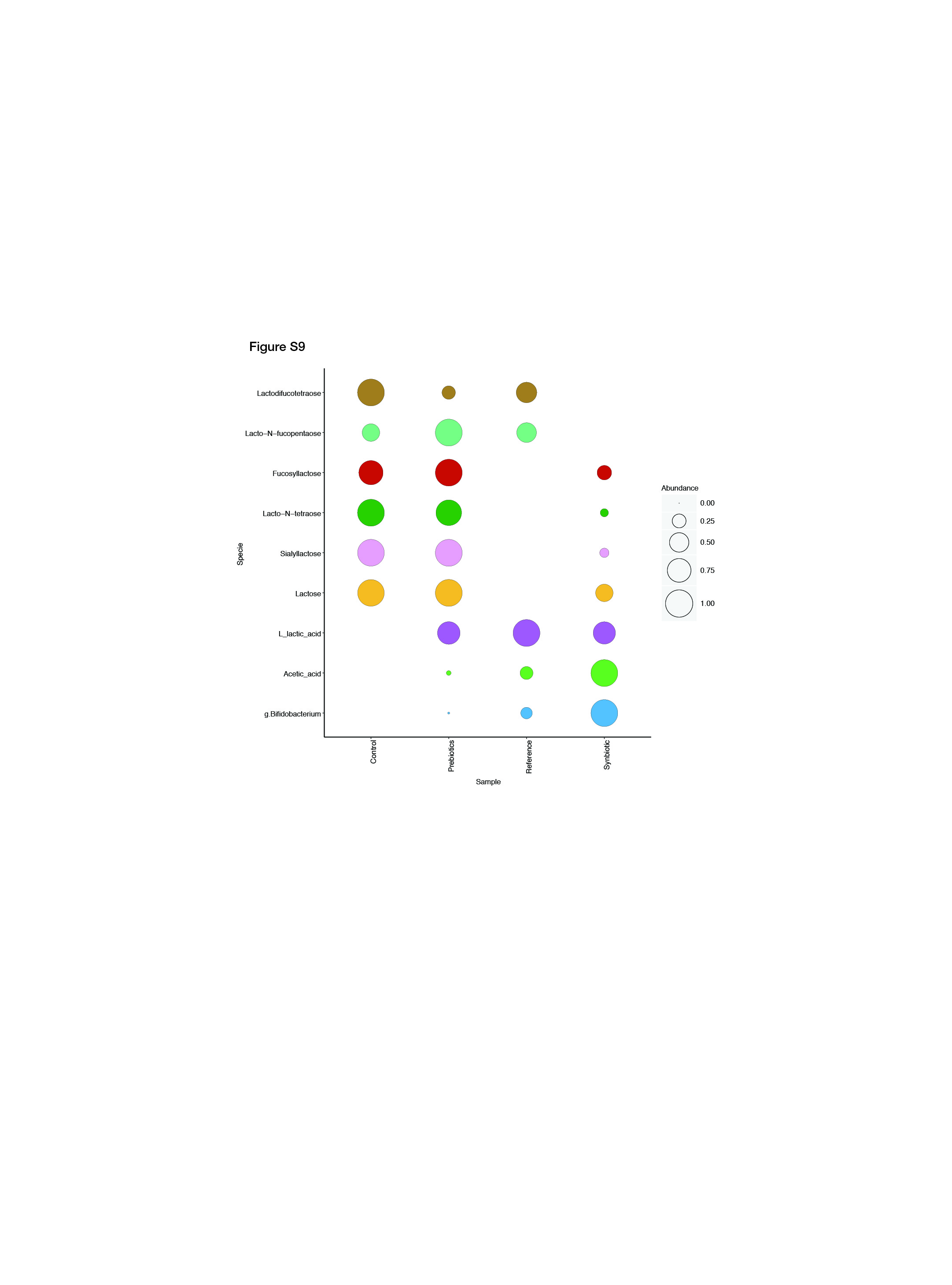


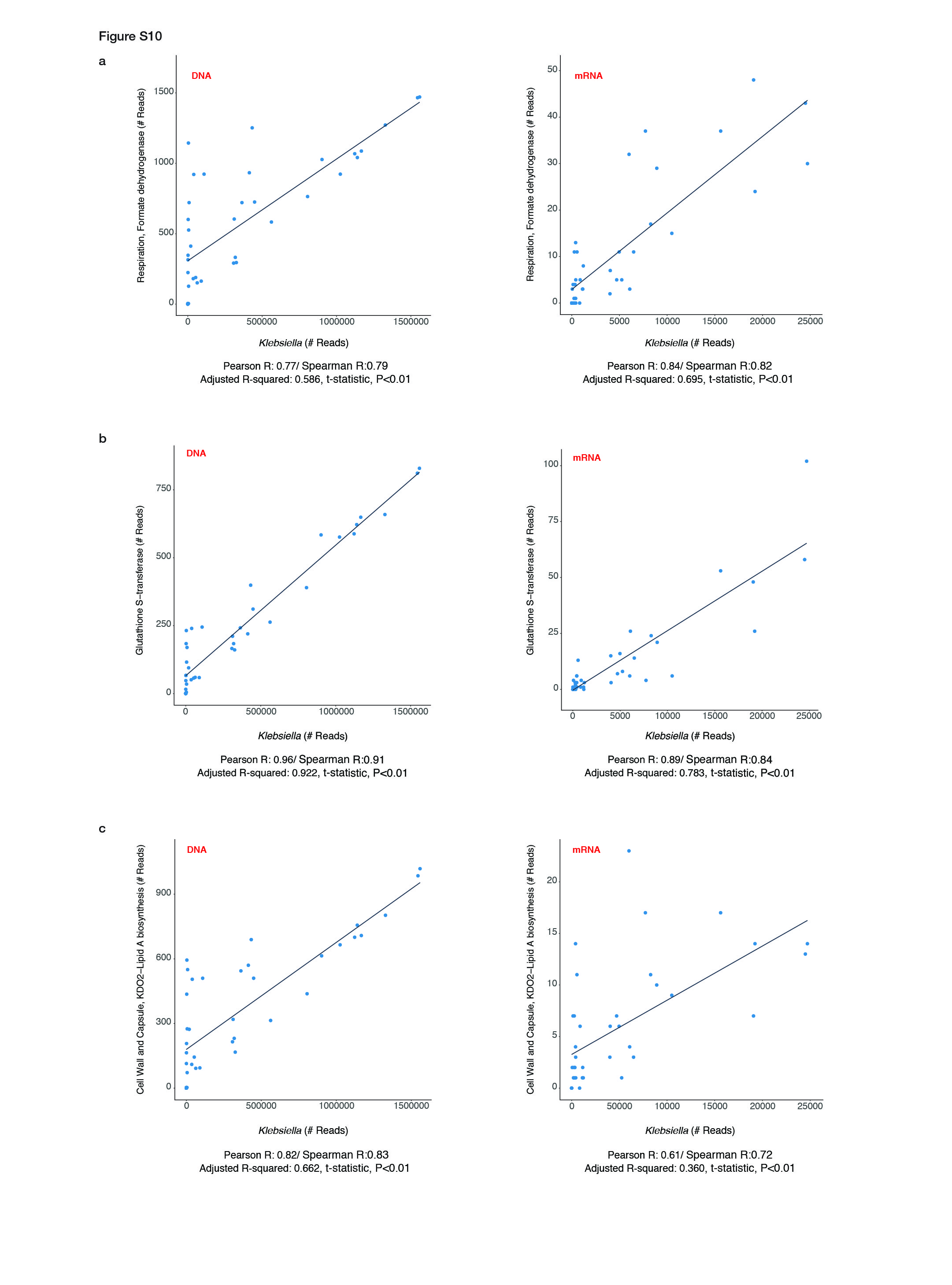


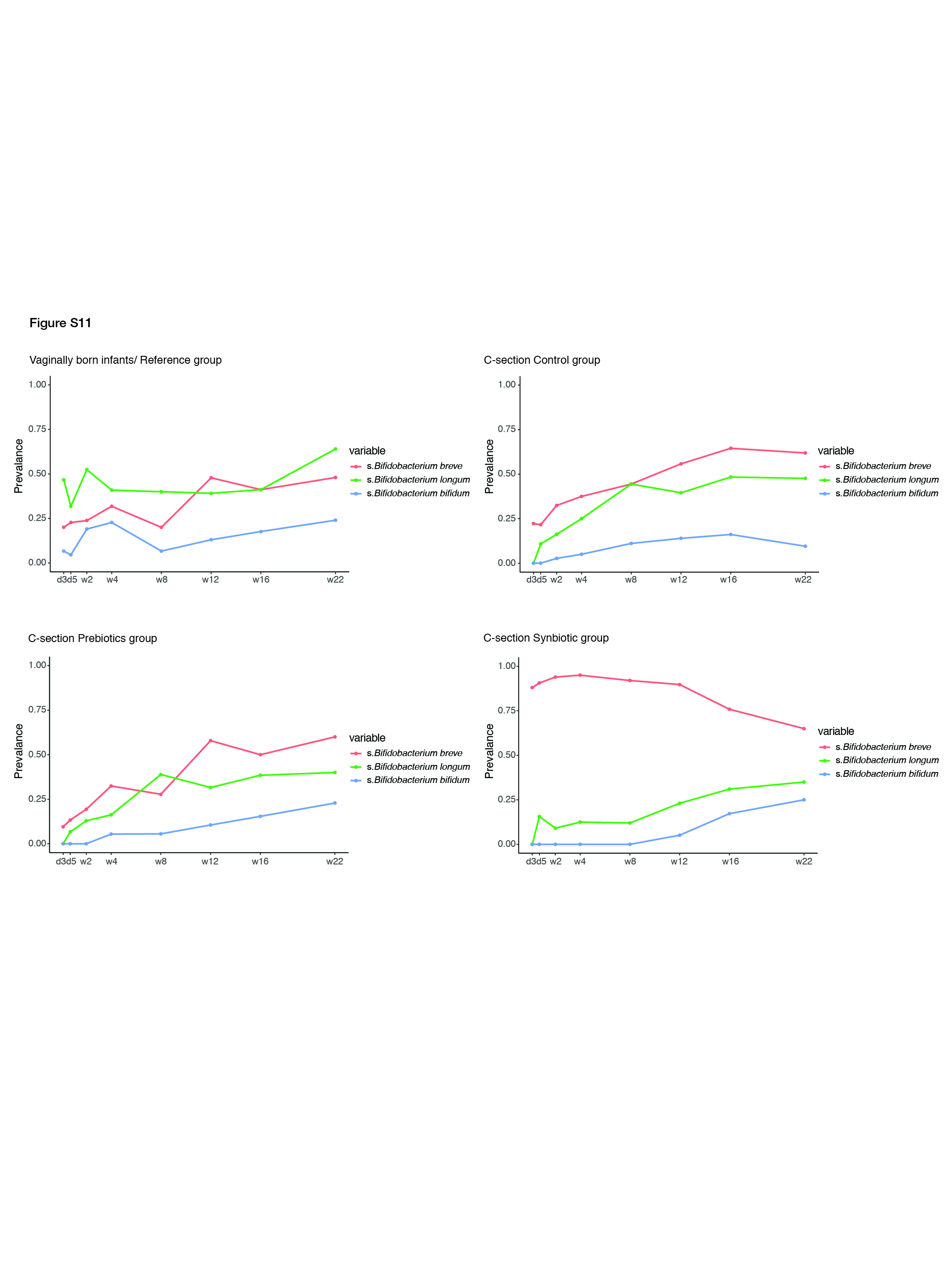


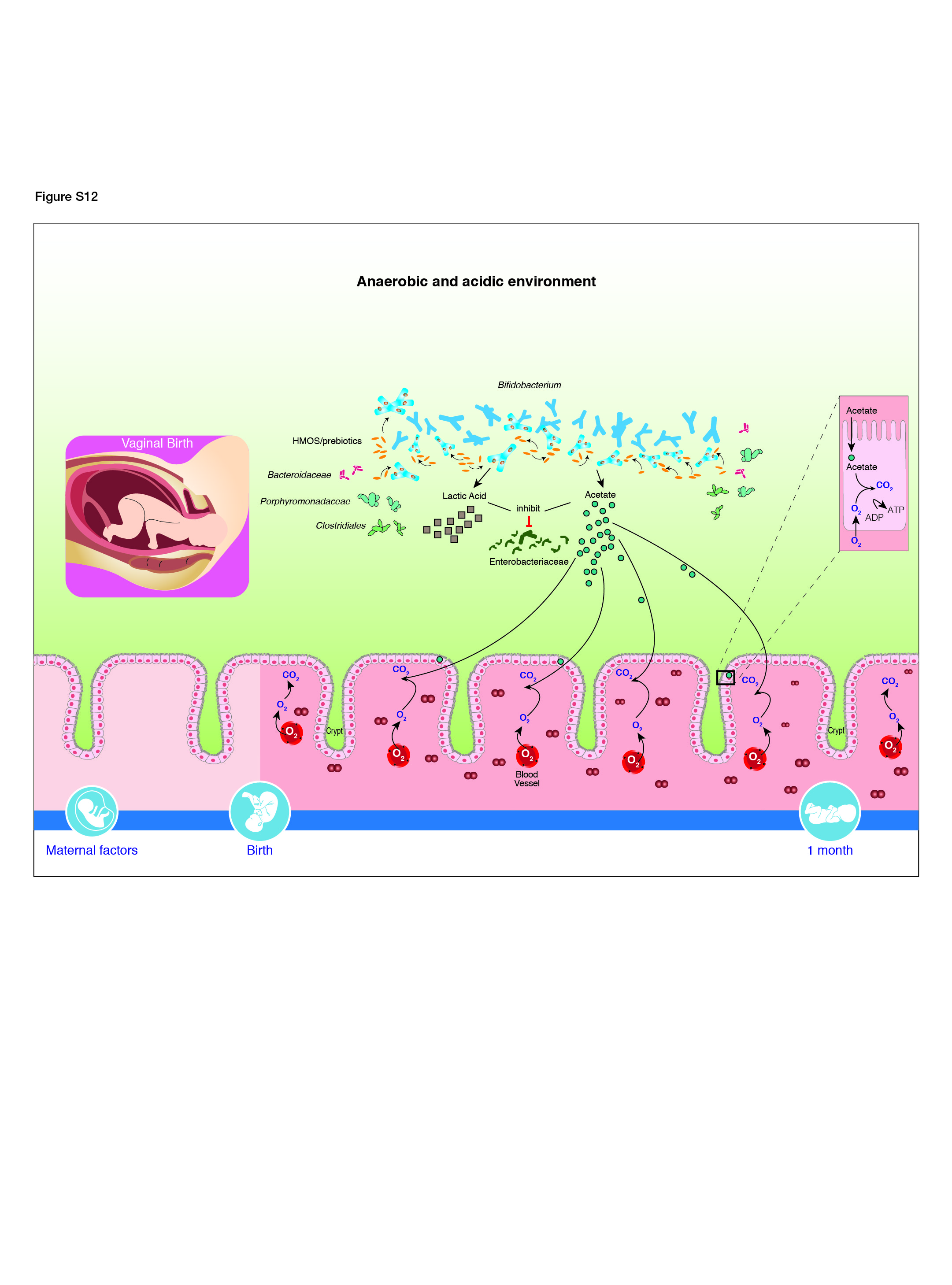


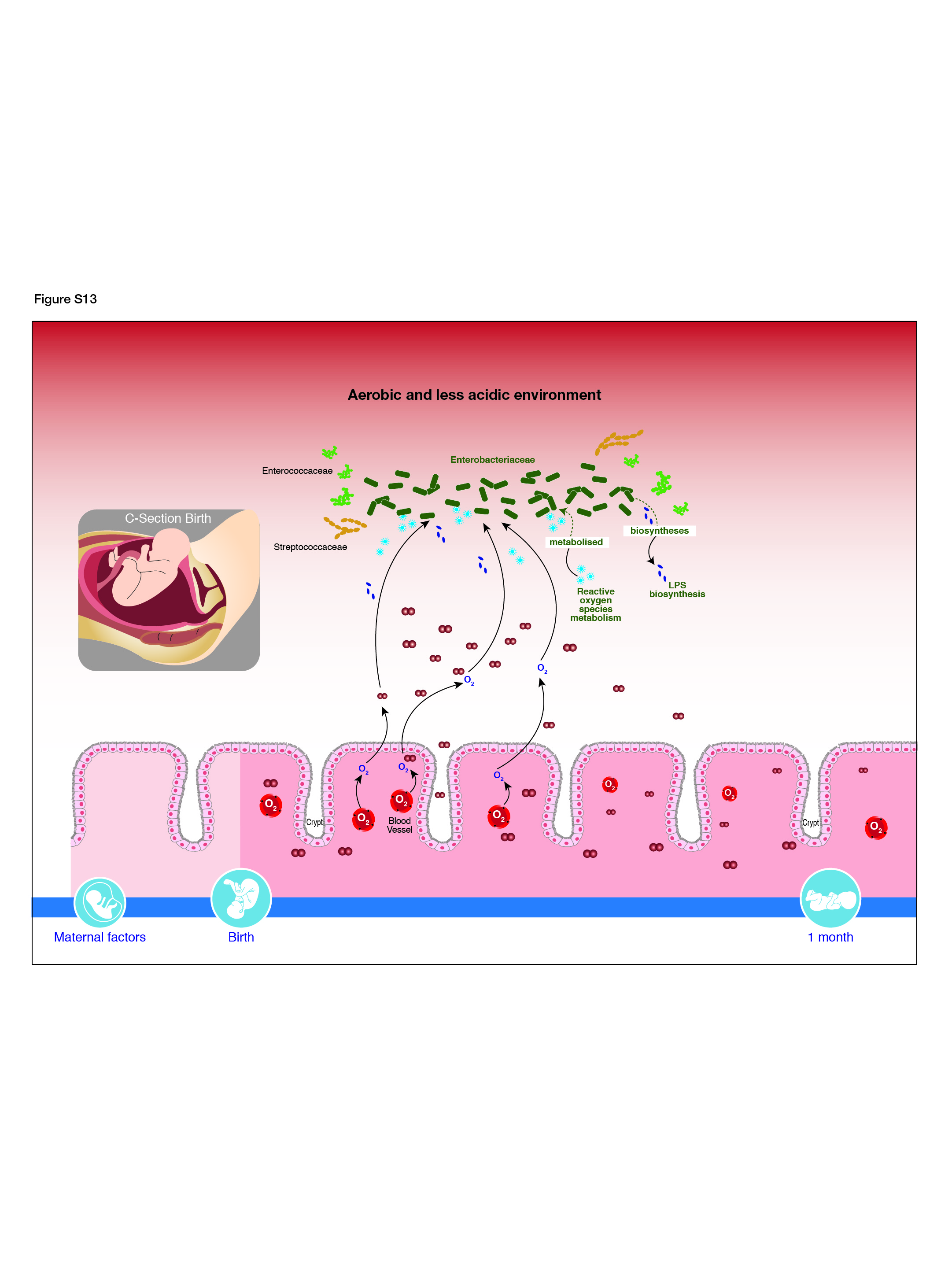

Supplement: Supplementary file 1 — Additional file 1: Figure S1. Prevalence of Bifidobacterium and Bacteroides per intervention group from day 3 till week 22. Figure S2a. Box-plots representing the distribution of samples along db-RDA1 at week 2. Non-parametric Mann-Whitney U tests P < 0.05, P < 0.01 and P < 0.001 are indicated by *, ** and ***, respectively. Figure S2b. Box-plots representing the distribution of samples along db-RDA1 at week 4. Non-parametric Mann-Whitney U tests P < 0.05, P < 0.01 and P < 0.001 are indicated by *, ** and ***, respectively. Figure S3. Relative abundance of Lactic Acid Bacteria (LAB). Stacked bar plot of the relative abundance of Lactic Acid Bacteria from day 3 till week 22. C: Control, P: Prebiotics, R: Reference, S: Synbiotic. Figure S4. Top 10 bacterial species identified among the 40 metagenomics samples from the four groups. Figure S5. Metabolic pathway derived from the metagenomics dataset depicting correlations with Bifidobacterium breve (blue) and Klebsiella (red). KEGG genes which exhibit Pearson correlation coefficients > 0.6 against the metagenomics reads assigned to the two species are mapped onto the KEGG metabolism pathway map. Figure S6. Metabolic pathway derived from the metatranscriptomics dataset depicting correlations with Bifidobacterium breve (blue) and Klebsiella (red). KEGG genes which exhibit Pearson correlation coefficients > 0.6 against the metatranscriptomic reads assigned to the two species are mapped onto the KEGG metabolism pathway map. Figure S7. Correlation between Bifidobacterium breve and HMOs metabolism. Linear model plot depicting correlation between Bifidobacterium breve and Lacto-N I Galacto-N-biose metabolism (SEED data). Figure S8. Heat map of lactose and HMO species present in the pooled samples for the synbiotic, prebiotics, control and reference groups (in triplicate). The colour scale is based on the row z-score. Figure S9. Abundance patterns of Bifidobacterium (16S rRNA), milk sugars, acetate and lactic acid as emerged fro [file 12866_2021_2230_MOESM1_ESM.docx]
